# When news is “written by artificial intelligence”: a systematic review of provenance and disclosure cues in journalism and their effects on credibility and trust

**DOI:** 10.3389/frai.2026.1815243

**Published:** 2026-05-05

**Authors:** Lorena Licenji, Julian Hoxha

**Affiliations:** 1 Liberal Arts Department, American University of the Middle East, Egaila, Kuwait; 2 College of Engineering and Technology, American University of the Middle East, Egaila, Kuwait

**Keywords:** artificial intelligence, automated journalism, credibility, disclosure labels, provenance cues, systematic review, trust

## Abstract

**Introduction:**

Artificial intelligence (AI) is increasingly embedded in journalism, yet audience responses may depend on both AI provenance, meaning who or what is presented as having written the story, and transparency cues that disclose AI use. This systematic literature review synthesises empirical studies examining how AI provenance cues and AI disclosure cues in journalism affect perceived credibility and trust.

**Methods:**

Following PRISMA 2020 and PRISMA-S, Scopus and Web of Science Core Collection were searched on 2 February 2026 for English-language, peer-reviewed journal articles and conference papers. Searches yielded 492 records. After deduplication and pre-screen exclusions, 290 records were screened at title/abstract level, and 47 studies with retrievable full texts were included. A structured narrative synthesis was conducted, guided by the Synthesis Without Meta-analysis (SWiM) guideline, to map study designs, cue operationalisations, outcome targets (message, source, outlet), and moderators.

**Results:**

Across heterogeneous designs, AI provenance cues were not associated with a consistent “AI penalty”: most extractable results indicated no difference between AI-attributed and human-attributed news, and observed effects were typically conditional on topic, baseline trust, outlet/source cues, and whether human oversight was signalled. Evidence on disclosure cues was limited (10 studies) and was dominated by null or conditional findings. Scepticism appeared more likely when disclosures implied full automation without accompanying accountability or oversight information.

**Discussion:**

A Cue–Inference–Target (CIT) framework is proposed to explain when AI cues shift epistemic-quality versus normative-legitimacy judgments. Future research should use factorial designs that separate provenance from disclosure and standardise reporting of cue wording, placement, and validated outcome measures.

## 1 Introduction

Artificial intelligence (AI) is becoming increasingly embedded in journalistic production, evolving from early forms of automated journalism, often described as algorithmic systems that transform structured data into narrative news with limited human intervention, toward contemporary generative AI capable of flexible, open-ended text production (Carlson, 2015; Diakopoulos, 2019; Danzon-Chambaud, 2021). Early newsroom applications of automation have been especially prominent in routine, data-to-text reporting contexts (Haim and Graefe, 2017). With the diffusion of large language models (LLMs), including widely used tools such as ChatGPT, news organisations and journalists are also experimenting with AI for editorial support tasks such as drafting and rewriting copy, summarising documents, generating headlines, and producing multiple versions of the same story for different audiences (Cools and Diakopoulos, 2024; Shi and Sun, 2024; Bykov and Kurushkin, 2025; Chen et al., 2025; Gavurova et al., 2024; Jeng et al., 2024; Moravec et al., 2024; Wu and Li, 2024). Because these newsroom applications rely on NLP-driven text generation and rewriting, understanding how audiences interpret provenance and disclosure cues is directly relevant to the responsible deployment and evaluation of natural language generation systems.

At the same time, generative AI’s ability to create convincing synthetic content has intensified concerns about misinformation, manipulation, and accountability in the public sphere. Synthetic political media can depict plausible events that never occurred, complicating audiences’ ability to assess authenticity and potentially undermining confidence in legitimate information sources (Chesney and Citron, 2019; Shin and Lee, 2022; Vaccari and Chadwick, 2020; Danry et al., 2025; Dobber et al., 2025; Kreps et al., 2022; Rae, 2024; Velásquez-Salamanca et al., 2025). For journalism, these risks are especially consequential because credibility and institutional trust are core resources: they shape whether audiences rely on news under uncertainty and whether journalistic institutions retain legitimacy as arbiters of public information.

Against this backdrop, transparency about AI use has become central to debates on responsible AI adoption in news. Newsrooms, platforms, and researchers increasingly discuss disclosure practices-such as AI bylines, labels, and disclosure/disclaimer statements-that signal when content is “AI-generated” or “AI-assisted” (Altay and Gilardi, 2024; Toff and Simon, 2025; Wittenberg et al., 2025). Yet transparency is not purely procedural; it is also a communicative cue that can shape audience judgments and potentially create trade-offs between ethical commitments and reputational outcomes (Krausová and Moravec, 2022).

A key conceptual challenge is that audiences can encounter AI involvement in news through two analytically distinct pathways that are often conflated. The first is AI provenance (authorship/production involvement): whether news is produced wholly or partly using automated, algorithmic, or AI-assisted processes (e.g., “automated journalism,” “algorithmic journalism,” “robot journalism,” or “machine-written/computer-generated news”). The second is AI disclosure (transparency cues): whether audiences are explicitly informed that AI was involved via a label, byline, or disclosure/disclaimer statement, regardless of whether the underlying process is AI-only or AI-assisted. Separating provenance from disclosure is essential because they plausibly operate through different mechanisms: provenance may trigger inferences about competence, appropriateness, and content quality, whereas disclosure cues may function as heuristics that shift attention from the message to the production agent and activate concerns about accountability and human oversight (Dietvorst et al., 2015; Sundar and Kim, 2019). To make this distinction concrete, provenance concerns who or what is presented as producing the journalistic content. For example, a study was treated as a provenance manipulation when it compared a news item attributed to a human journalist versus an AI system, or when it compared AI-produced versus human-produced versions of a story. Disclosure, by contrast, concerns whether audiences are explicitly told that AI was involved, for example through an “AI-generated” label, an AI byline, or a disclosure statement attached to otherwise comparable content. In some studies, both pathways were present at the same time, such as factorial designs that varied both the apparent authorship/production source of the story and the presence or wording of an AI disclosure. In this review, such cases were not collapsed into a single category; instead, they were coded as combined provenance-plus-disclosure designs and interpreted separately so that the effect of disclosure was not conflated with the effect of provenance.

These pathways also matter because journalism research treats trust and credibility as related but distinct constructs, and the studies included in this review did not operationalise them in a fully uniform way. Trust is commonly defined as a willingness to rely on an actor or institution under conditions of uncertainty and vulnerability (Mayer et al., 1995) and has been operationalised in news contexts as multidimensional confidence in journalistic processes such as selection, accuracy, and evaluation (Kohring and Matthes, 2007). Credibility, while overlapping with trust, is typically conceptualised as perceived believability and is often grounded in judgments of expertise and trustworthiness; it can be assessed at multiple levels (message/article credibility vs. source/outlet credibility) (Flanagin and Metzger, 2007; Metzger and Flanagin, 2013; Barrolleta and Sandoval-Martín, 2024; Gong, 2023; Heiselberg et al., 2022; Jia and Johnson, 2021; Kim and Kim, 2020; Tewari et al., 2021). Across the evidence base, studies varied in both the target of evaluation and the measurement instruments they used, ranging from message-level credibility assessments to source- or outlet-level trust judgments, and from validated multi-item scales to *ad hoc* indicators. This matters for the review’s conclusions because studies may appear to differ not only because of AI provenance or disclosure cues themselves, but also because they are assessing different evaluative targets: message-level credibility can remain stable even when trust in the producing actor, outlet, or production process is more contested. Accordingly, the review distinguishes between constructs, targets of evaluation, and measurement choices when synthesising findings.

Empirical findings on AI in journalism are mixed and sometimes difficult to reconcile across designs and contexts. Audience studies on automated journalism frequently report small, context-dependent differences in credibility between machine-written and journalist-written news (Barrolleta and Sandoval-Martín, 2024; Gong, 2023; Heiselberg et al., 2022; Jia and Johnson, 2021; Kim and Kim, 2020; Tewari et al., 2021) rather than a uniform “AI penalty,” especially for routine or data-driven reporting (Graefe et al., 2018; Haim and Graefe, 2017; Tandoc et al., 2020; Wölker and Powell, 2021). At the same time, a newer stream of research suggests that disclosure itself can sometimes depress perceived accuracy, credibility, or trust, creating what has been described as a transparency- or disclosure-related penalty, though results vary by cue design and context (Altay and Gilardi, 2024; Jia et al., 2024; Liu et al., 2023; Toff and Simon, 2025).

The literature is further complicated by heterogeneity in disclosure design and study context. AI cues vary in wording (“AI-generated” vs. “AI-assisted”), framing (neutral transparency vs. warning-like disclaimers), prominence and placement (headline tags, bylines, footers), and whether they communicate human oversight. Effects may also depend on topic sensitivity, modality, and audience characteristics such as AI familiarity and baseline trust in journalism (Haim et al., 2025; Wittenberg et al., 2025). Moreover, relevant evidence is distributed across journalism studies, communication, HCI, and behavioural research (Govers et al., 2025; Schulz et al., 2022), where trust and credibility are operationalised differently, limiting straightforward comparison across studies.

For these reasons, a systematic synthesis is needed that focuses specifically on news/journalism, clearly separates AI provenance from AI disclosure cues (labels, bylines, disclosures/disclaimers), and consolidates evidence on their effects on audience trust and perceived credibility.

Aligning with the constructs captured by the Scopus and Web of Science queries used in this review, the synthesis maps and integrates empirical audience research on automated/algorithmic and generative AI in news, including studies explicitly examining AI-generated/AI-assisted content and AI disclosure practices, to clarify when AI involvement undermines trust and credibility, when effects are neutral or contingent, and which moderators are most consistently supported by current evidence.

### 1.1 Aim and research questions

This systematic literature review synthesises empirical audience research examining how artificial intelligence (AI) involvement in journalistic news production, and the disclosure of that involvement, shapes perceived credibility and trust. The review is scoped to news/journalism contexts in which AI involvement is described as automated/algorithmic/robot journalism, machine-written/computer-generated news, or more recent generative AI/LLM applications (e.g., “AI-generated,” “AI-assisted,” or ChatGPT) (Clerwall, 2014; Graefe et al., 2018; Haim and Graefe, 2017; Shi and Sun, 2024).

Consistent with the logic of the final database queries (Table 1), the review treats two analytically distinct mechanisms as central. The first is provenance (authorship/production involvement): whether a news item is produced wholly or partly with AI (e.g., AI-authored or AI-assisted) versus produced by human journalists (Clerwall, 2014; Leuppert et al., 2025; Tandoc et al., 2020). The second is disclosure (transparency cues): whether audiences are explicitly informed that AI was involved via cues captured by the search strategy: labels, AI bylines, and disclosure/disclaimer statements, regardless of whether the underlying content differs (Altay and Gilardi, 2024; Bien-Aimé et al., 2025; Toff and Simon, 2025; Wittenberg et al., 2025). Distinguishing provenance from disclosure is important because authorship information may shape inferences about competence or appropriateness, whereas disclosure cues may operate as heuristics that elevate scepticism or generate transparency-related penalties even when content is otherwise comparable (Dietvorst et al., 2015; Schilke and Reimann, 2025; Sundar and Kim, 2019). In NLP terms, provenance cues function as authorship-attribution signals for machine-generated or machine-assisted text, while disclosure cues function as transparency labels about the role of a language model in text production.

**Table 1:** Database search strings used in the main search and sensitivity check (2 February 2026).

Database and query type	Search string syntax	Limits applied
SCOPUS Main Advanced Search	TITLE-ABS-KEY((news OR journalis*) AND (trust* OR credib*) AND (“automated journalism” OR “algorithmic journalism” OR “robot journalism” OR “algorithm authorship” OR “computer generated news” OR “machine written news” OR “AI generated” OR “AI assisted” OR “generative AI” OR ChatGPT OR ((label* OR byline* OR disclos* OR disclaimer*) W/5 AI)))	PUBYEAR > 2013 AND PUBYEAR < 2027
SCOPUS Sensitivity check	TITLE-ABS-KEY((news OR journalis*) AND (ChatGPT OR “generative AI” OR “AI generated” OR “AI assisted” OR “automated journalism” OR “algorithmic journalism”) AND (disclos* OR label* OR byline* OR disclaim*))	PUBYEAR > 2013 AND PUBYEAR < 2027
WEB OF SCIENCE Main Advanced Search	TS = ((news OR journalis*) AND (trust* OR credib*) AND (“automated journalism” OR “algorithmic journalism” OR “robot journalism” OR “automated news” OR “machine written news” OR “computer generated news” OR “AI generated” OR “AI assisted” OR “generative AI” OR ChatGPT OR ((label* OR byline* OR disclos* OR disclaim*) NEAR/5 (“artificial intelligence” OR “AI”))))	PUBYEAR > 2013 AND PUBYEAR < 2027
WEB OF SCIENCE Sensitivity search	TS = ((news OR journalis*) AND (ChatGPT OR “generative AI” OR “AI generated” OR “AI assisted” OR “automated journalism” OR “algorithmic journalism”) AND (disclos* OR label* OR byline* OR disclaim*))	PUBYEAR > 2013 AND PUBYEAR < 2027

The outcomes of interest are *trust* and *credibility,* which are related but not identical. Trust is commonly defined as willingness to rely on an actor or institution under conditions of uncertainty and vulnerability (Mayer et al., 1995) and, in news contexts, is often conceptualised as confidence in journalistic processes such as selection, accuracy, and assessment (Kohring and Matthes, 2007). Credibility refers to perceived believability of the message and/or its source and is frequently operationalised using message- or source-credibility measures and validated credibility scales (Appelman and Sundar, 2016; Metzger and Flanagin, 2013). Because studies vary in whether they assess article-level credibility, journalist/source credibility, or outlet-level trust, the review distinguishes between targets of evaluation and measurement choices when synthesising findings.

The review is guided by the following research questions:

RQ1 (AI provenance/authorship effect). In empirical audience studies of journalism/news, what is the effect of AI provenance, news described as AI-generated, AI-assisted (human-AI collaboration), or produced via automated/algorithmic/robot journalism, on perceived credibility and trust (message/article and source/outlet targets), relative to human/journalist authorship?

RQ2a (AI disclosure cue main effect). When AI involvement is explicitly disclosed, how do disclosure cues (labels, AI bylines, and disclosure/disclaimer statements) affect perceived credibility and trust compared with no AI disclosure and/or human-origin cues?

RQ2b (AI disclosure cue design contingencies). What evidence exists that cue characteristics (e.g., “AI-generated” vs. “AI-assisted” wording, framing, placement/prominence, and stated human oversight) condition the effects of AI disclosure cues?

RQ3 (moderators and measurement). Which study-level moderators beyond disclosure-cue design (e.g., topic/stakes, modality, outlet context, cultural setting, and audience characteristics such as baseline news trust and AI familiarity) and measurement choices (credibility vs. trust; message vs. source/outlet targets; validated scales vs. *ad hoc* items) help explain heterogeneity and limit comparability across studies?

## 2 Methods

This systematic literature review was designed and reported in accordance with the PRISMA 2020 statement (Page et al., 2021) and PRISMA-S for transparent reporting of search strategies (Rethlefsen et al., 2021). A review protocol was prepared prior to screening to prespecify the review questions, eligibility criteria, information sources, search strategies, screening procedures, and synthesis approach, consistent with recommendations to reduce ad hoc decision-making and selective reporting (Moher et al., 2015; Page et al., 2021).

**Figure 1:**
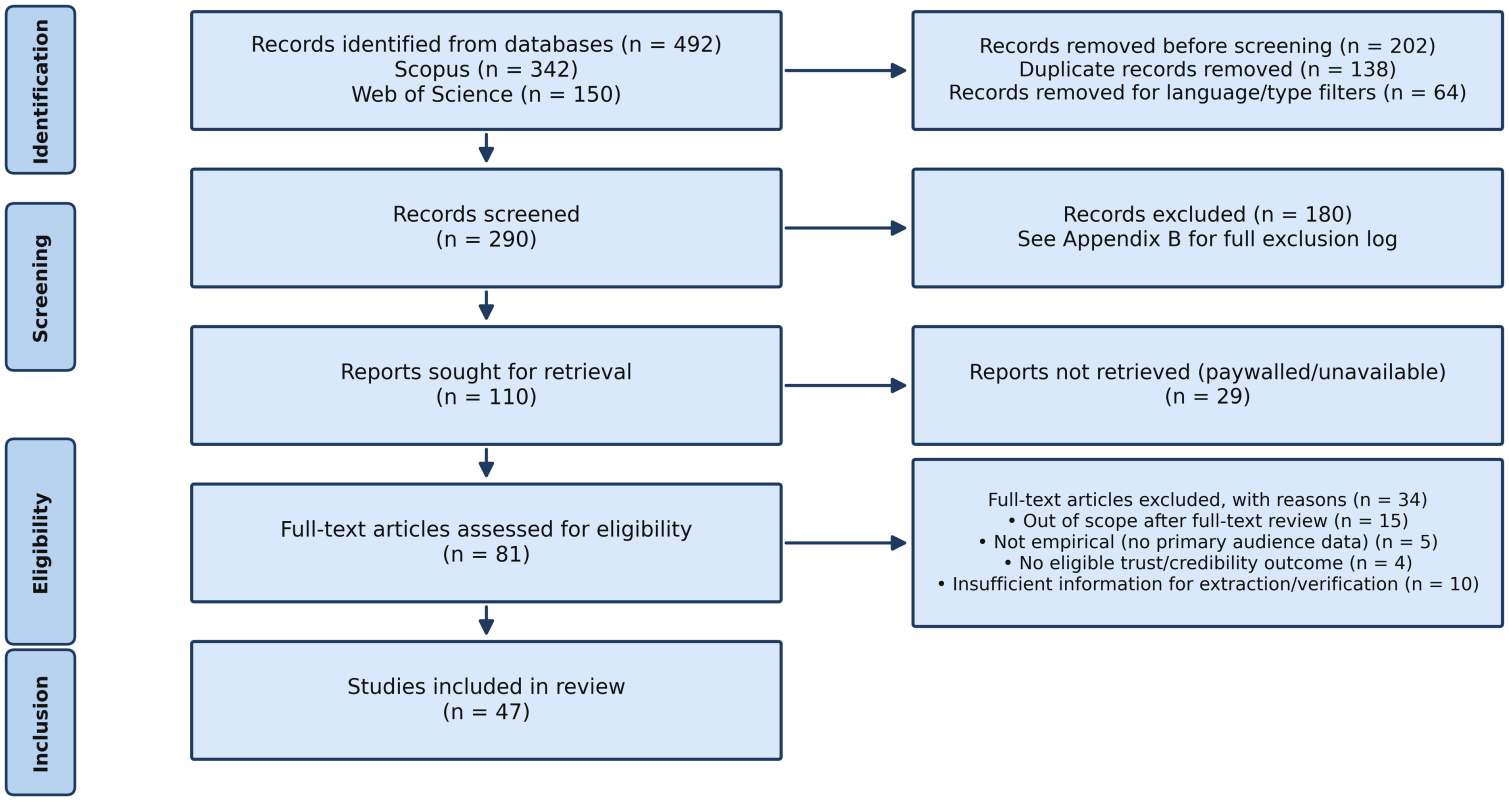
PRISMA 2020 flow diagram for the systematic review of AI-generated news and public trust (full-text synthesis set).

Screening, eligibility assessment, data extraction, and synthesis were conducted by the first author; the second author contributed to protocol development, interpretation of findings, and manuscript revision. To mitigate the risk of selection and extraction error associated with single-reviewer processes, inclusion/exclusion decision rules were prespecified in the protocol, screening and extraction were documented in structured logs, and ambiguous cases were revisited during full-text assessment and synthesis. Consistent with the review’s reliability aim, study-level extraction and synthesis were conducted only for reports with retrievable full-text PDFs (see Figure 1); records that appeared eligible but could not be retrieved were documented as “reports not retrieved” and were not coded. A generative AI tool (ChatGPT, GPT-5.2 Pro, OpenAI) was used solely for language editing; it was not used to automate screening, extraction, or synthesis decisions.

Following PRISMA-S, the search strategy was structured using concept blocks aligned with the review framework and the final database strings: (1) journalism/news context, (2) trust/credibility outcomes, and (3) AI involvement in news production and/or AI disclosure cues (Rethlefsen et al., 2021). Within each block, synonyms were combined with OR; blocks were combined with AND. Truncation (e.g., journalis*, credib*, trust*) was used to increase retrieval breadth. Proximity operators were used to connect disclosure-cue terms to AI terms (Scopus: W/5; Web of Science: NEAR/5) to reduce retrieval of generic disclosures unrelated to AI while retaining sensitivity for relevant disclosure variants.

To reduce the risk of missing disclosure-focused studies that examine AI labelling/bylines/disclaimers in news contexts but do not include “trust” or “credibility” language in searchable fields, a supplementary disclosure-focused sensitivity search was conducted. This sensitivity search retained the news/journalism, AI, and disclosure blocks but removed the trust/credibility block, and its results were deduplicated against the main search results and screened using the same eligibility criteria (Rethlefsen et al., 2021).

### 2.1 Eligibility criteria

Eligibility criteria were defined using PICOS logic (Population, Exposure, Comparator, Outcomes, Study design) adapted to empirical audience research in journalism/news (Higgins et al., 2024).

*Population*: Eligible studies involved human participants (e.g., news audiences) exposed to journalistic or news stimuli. Samples could be drawn from online panels, laboratory/classroom settings, or field contexts. Studies were excluded if they were purely technical/computational (e.g., system descriptions, detection/classification benchmarks, model performance evaluations) and did not include human perception, evaluation, or judgment measures.

*Exposure (phenomenon of interest)*: AI involvement in journalism was operationalised in two ways, consistent with the final search strings:

Provenance/authorship (AI in production): Studies were eligible if they examined whether news was produced wholly or partly using AI (e.g., automated/algorithmic/robot journalism, machine-written/computer-generated news, AI-generated or AI-assisted news) compared with human/journalist-produced news.

*Disclosure cues (explicit AI indication)*: Studies were eligible if they examined whether audiences were explicitly informed that AI was involved via cues captured by the search strategy (labels, AI bylines, and/or disclosure/disclaimer statements indicating AI involvement; e.g., “AI-generated,” “AI-assisted”). Disclosure could be examined as a standalone manipulation (holding content constant) or combined with provenance manipulations in factorial designs.

To maintain a coherent scope aligned with the query terms (news OR journalis*), stimuli had to be framed as journalism/news (e.g., news headlines, news articles, news reports, news posts, or news-style segments). Studies centred on advertising/marketing, e-commerce, customer service, or non-news organisational communication were excluded unless the stimulus was explicitly presented as journalistic news.

*Comparator*: Eligible studies included at least one relevant comparator condition such as: AI-authored/AI-assisted vs. journalist-authored news; AI disclosure cue vs. no disclosure and/or a human-origin cue; and/or comparisons among disclosure designs (e.g., “AI-generated” vs. “AI-assisted” wording; warning-like vs. neutral framing; prominent vs. subtle placement; disclosure with vs. without stated human oversight).

*Outcomes:* To align with the review’s scope and the outcome block in the main query (trust* OR credib*), each included study reported at least one outcome related to perceived credibility and/or trust, operationalised quantitatively (e.g., message/article credibility scales, source/outlet credibility, trust indices) and/or qualitatively (e.g., interview/focus group themes about perceived trustworthiness or credibility). Trust was conceptualised as a willingness to rely on an actor or institution under conditions of uncertainty and vulnerability (Mayer et al., 1995) and, in news contexts, often reflects confidence in journalistic processes such as accuracy and assessment (Kohring and Matthes, 2007). Credibility was conceptualised as perceived believability and evaluations of a message or source’s trustworthiness and/or expertise and is commonly operationalised using message/source credibility measures (Appelman and Sundar, 2016; Flanagin and Metzger, 2007; Metzger and Flanagin, 2013). Studies were excluded if they reported no trust/credibility outcomes (e.g., usability outcomes only). For synthesis, direction-of-effect coding was applied when studies reported extractable quantitative comparisons; qualitative evidence was synthesised narratively to identify mechanisms and moderators.

*Study design*: The review included empirical audience research using any design (experiments, surveys, qualitative interviews/focus groups, or mixed methods), provided that the study examined AI provenance/authorship and/or AI disclosure cues in a news/journalism context and reported trust/credibility-related outcomes.

*Publication characteristics:* The review primarily targeted English-language, peer-reviewed journal articles and peer-reviewed conference papers/proceedings within the prespecified publication window used in the searches (as indexed on the search date). Two accessible full-text records were available as working papers/preprints but were retained when they reported empirical audience evidence and provided sufficient methodological detail for extraction; these records are flagged in the dataset and interpreted cautiously in the synthesis. Where records appeared eligible but full texts could not be retrieved after reasonable efforts, they were documented as “reports not retrieved” in the PRISMA record list but were not included in full-text extraction and synthesis to protect the reliability of study-level coding.

### 2.2 Information sources and search strategy

Searches were conducted in Scopus and Web of Science Core Collection, which provide broad multidisciplinary indexing and are widely used in systematic evidence syntheses where relevant studies span multiple fields (e.g., journalism studies, communication, HCI) (Gusenbauer and Haddaway, 2020). Using more than one database is recommended to reduce retrieval bias and improve coverage because no single index is comprehensive across outlets and disciplines (Bramer et al., 2017; Higgins et al., 2024).

The main database searches were executed on 2 February 2026. Searches were applied to title/abstract/keyword-indexed fields (Scopus: TITLE-ABS-KEY; Web of Science: TS). Limits for language (English) and document types (journal articles and conference proceedings/papers) were applied using database filters where available; after export and merging, records were checked again and any remaining non-eligible language/type items were removed prior to screening (see Figure 1). No study-design filters were applied at the search stage to maximise sensitivity; design and outcome criteria were enforced during screening (Higgins et al., 2024).

The final main search strings combined three blocks:

- Journalism/news context: (news OR journalis*)
- Outcomes: (trust* OR credib*)
- AI involvement and/or disclosure cues: terms covering automated/algorithmic/robot journalism, machine-written/computer-generated news, generative AI/ChatGPT, and disclosure cues (labels/bylines/disclosures/disclaimers) connected to AI using proximity operators.

A supplementary minimal sensitivity search was also conducted in each database by retaining the journalism/news, AI and disclosure blocks while removing the trust/credibility block. This step was included to mitigate the risk of missing disclosure-relevant studies that do not use trust/credibility terminology in searchable fields (Rethlefsen et al., 2021). The database-specific strings (main and sensitivity) are reported in Table 1.

### 2.3 Study selection

Study selection followed PRISMA 2020 guidance (Page et al., 2021) and proceeded in three stages: (1) record management and deduplication, (2) title/abstract screening, and (3) full-text eligibility assessment.

*Record management and deduplication*: Records retrieved from Scopus and Web of Science (main and sensitivity searches) were exported and merged into a single screening library. Duplicates were removed prior to screening using DOI matching where available and, when DOI was missing, by a standardised title and publication year match. This approach is consistent with established deduplication recommendations for systematic reviews (Bramer et al., 2016) (Table 2).

**Table 2:** Evidence map of included full-text studies (*N =* 47): exposure type × outcome domain.

Exposure type	Credibility	Trust	Credibility + Trust	Unclear	Row total
Provenance/authorship	17 (36.2%)	5 (10.6%)	14 (29.8%)	0 (0.0%)	36 (76.6%)
Disclosure cues	0 (0.0%)	0 (0.0%)	1 (2.1%)	0 (0.0%)	1 (2.1%)
Combined (provenance + disclosure)	5 (10.6%)	1 (2.1%)	2 (4.3%)	1 (2.1%)	9 (19.1%)
Unclear	0 (0.0%)	0 (0.0%)	1 (2.1%)	0 (0.0%)	1 (2.1%)
Total	22 (46.8%)	6 (12.8%)	18 (38.3%)	1 (2.1%)	47 (100.0%)

Cells show counts with percent of total *N* = 47 in parentheses.

*Full-text screening*: Retrieved full texts were assessed for eligibility using prespecified criteria: (a) the stimulus was framed as journalism/news, (b) an eligible AI exposure was present (provenance/authorship and/or disclosure cue), (c) an appropriate comparator or clear qualitative contrast was included, and (d) at least one credibility and/or trust-related outcome was reported (quantitative and/or qualitative). Reasons for exclusion at the full-text stage were recorded (out of scope for the final synthesis; not an empirical audience study; no eligible trust/credibility outcome; insufficient information for extraction/verification) in accordance with PRISMA 2020 reporting expectations (Page et al., 2021).

Title/abstract screening and full-text eligibility assessment were conducted by the first author using prespecified criteria and a structured screening log. To maximise sensitivity, any record coded as include or uncertain at the title/abstract stage advanced to full-text retrieval attempts and/or assessment when available. Searches identified 492 records (Scopus *N =* 342; Web of Science *N =* 150). After deduplication (*N =* 138) and pre-screen exclusions based on language/document type (*N =* 64), 290 records were screened at title/abstract and 180 were excluded. Most exclusions reflected absent trust/credibility outcomes or technical/system-focused records without human participants (see Appendix B for the full exclusion log). Full texts were sought for 110 reports; 29 were not retrievable through institutional subscriptions or open-access channels, leaving 81 full texts assessed. Of these, 34 reports were excluded at full-text review. The excluded full-text reports are listed individually in Appendix B, together with their primary exclusion reason. The final synthesis included 47 studies with retrievable full-text PDFs (Figure 1). The PRISMA master list and screening/exclusion log, including the 34 reports excluded at full-text review and their primary exclusion reasons, are provided in Appendix B; the coded extraction dataset is provided in Appendix A.

### 2.4 Data extraction and coding

Data extraction was conducted using a structured codebook developed for this review and pilot-tested on a subset of included studies to refine definitions and ensure consistent interpretation before full extraction (Higgins et al., 2024). The unit of analysis was the published report (journal article or conference paper/proceedings). Where a single report described multiple empirical studies (e.g., separate experiments or distinct samples), all relevant provenance/disclosure manipulations and trust/credibility findings were coded within the same record; for descriptive summaries, the largest reported participant sample size per report was used as a conservative descriptor. The codebook captured: (a) bibliographic descriptors (year, outlet type), (b) design and sample characteristics (design type, recruitment method, sample size, country/region when reported, and relevant participant characteristics such as AI familiarity when available), and (c) stimulus characteristics (news format, modality, and topic area). Topic area was coded using journalism-relevant categories (e.g., politics, health/science, economy/business, crime, routine/service news) to support moderator mapping. Country/region was extracted not only descriptively but because geographical/cultural setting was treated as a potential moderator in RQ3; where studies reported relevant cultural, political, or media-system context, this information was considered during the narrative synthesis.

Coding of AI involvement aligned with the final query terms and the review framework. Studies were coded as examining: (i) provenance/authorship, (ii) disclosure cues, or (iii) combined designs. Provenance coding captured how AI involvement was operationalised (e.g., automated/algorithmic/robot journalism; AI-generated; AI-assisted/LLM-assisted writing; human-AI collaboration) and whether conditions implied content differences or attribution-only differences (i.e., identical content with different authorship/provenance information). Disclosure coding captured whether otherwise comparable content was presented with an explicit AI cue and, when present, the cue’s characteristics: cue type (label, AI byline, and/or disclosure/disclaimer statement), wording (e.g., “AI-generated” vs. “AI-assisted”), framing (neutral informational vs. warning-like language), placement/prominence, timing (pre-exposure vs. concurrent vs. post-exposure), and whether the cue communicated human oversight or editorial accountability.

Outcome extraction focused on perceived credibility and/or trust and recorded: the target of evaluation (message/article-level, journalist/source-level, outlet/newsroom-level, and platform-level where applicable), construct labels used by authors (e.g., credibility, accuracy, believability, objectivity/bias, trustworthiness, expertise, trust), and measurement properties (single-item vs. multi-item scales; reliability when reported) (Appelman and Sundar, 2016; Metzger and Flanagin, 2013). An effect was treated as extractable when a directionally interpretable quantitative comparison between an AI-cued condition and an eligible comparator (e.g., human-authored or no-disclosure condition) was reported with sufficient statistical detail (e.g., group means/SDs, test statistics and *p*-values, or model coefficients with uncertainty) to determine the direction of the effect. Where reporting was insufficient to determine direction (or where evidence was qualitative only), the outcome was coded as not extractable and was synthesised narratively.

For qualitative studies, extraction focused on themes related to trust/credibility judgments, perceived mechanisms, and expectations regarding transparency and accountability. These studies were not excluded from the review, but they were treated differently from studies that reported extractable quantitative contrasts. Specifically, qualitative studies were appraised using the qualitative component of the MMAT framework and were synthesised narratively to identify interpretive frames, perceived mechanisms, and contextual conditions shaping audience responses to AI provenance and disclosure cues. They therefore informed the review’s explanation of why effects varied across contexts, rather than contributing to direction-of-effect counts.

Extraction was completed using the structured codebook. Where reporting was incomplete or ambiguous (e.g., multiple outcomes or complex factorial designs), the full text was re-consulted and coding decisions were documented to reduce interpretive drift (Higgins et al., 2024). The final coded extraction dataset for the included full-text studies is provided in Appendix A.

### 2.5 Risk of bias and quality appraisal

Given heterogeneity in study designs and reporting practices across the included evidence base, methodological quality and risk of bias were appraised to support cautious interpretation rather than to impose a numerical exclusion threshold. Quality appraisal was conducted by the first author using MMAT guidance version 2018, which is designed to enable consistent critical appraisal across qualitative, randomised, non-randomised, quantitative descriptive, and mixed-methods study designs (Hong et al., 2018). The second author contributed to interpretation of the appraisal implications during manuscript revision.

MMAT was selected because the evidence base includes experimental audience studies, survey designs, and qualitative/mixed-methods studies relevant to AI provenance and disclosure in journalism, and a unified appraisal framework facilitates comparison across designs without requiring multiple toolsets. To strengthen interpretability, MMAT judgments were applied with explicit attention to validity concerns that are particularly salient in this literature (e.g., clarity and plausibility of provenance manipulations; specificity and prominence of labels/bylines/disclosures/disclaimers; and construct validity of trust/credibility outcomes). Where design-specific appraisal required additional conceptual guidance, criteria were interpreted in a manner consistent with established risk-of-bias frameworks for randomised and non-randomised evidence (Sterne et al., 2016; Sterne et al., 2019).

Appraisal focused on criteria most relevant to interpretability and, where applicable, causal inference:

- Experimental designs: adequacy of randomisation/assignment procedures, baseline comparability, clarity of AI provenance and/or disclosure manipulations, use and interpretation of manipulation checks (when reported), attrition and missing-data handling, and transparency of analytic decisions.
- Survey and quantitative descriptive designs: recruitment quality (including nonresponse considerations where reported), measurement quality, and plausibility/handling of confounding when causal interpretations were implied.
- Qualitative components: coherence of sampling, transparency of analytic procedures, and grounding of interpretations in the presented evidence.

MMAT judgments were used to contextualise confidence in findings and to support cautious interpretation rather than to apply a numerical exclusion threshold (Hong et al., 2018). Study-level appraisal notes are provided in Appendix E.

### 2.6 Synthesis approach

A meta-analysis was not specified as the primary synthesis method because included studies varied substantially in: (a) how AI involvement was operationalised (e.g., automated/algorithmic/robot journalism; AI-generated vs. AI-assisted; generative AI/ChatGPT framing), (b) how disclosure cues were implemented (labels, AI bylines, disclosure/disclaimer statements; wording, framing, and placement), (c) the level and target of outcomes (message/article credibility vs. journalist/source or outlet/newsroom trust), and (d) the availability and comparability of effect-size reporting. In systematic reviews where quantitative pooling is not appropriate or not feasible, transparent reporting of alternative synthesis methods is recommended (Campbell et al., 2020; Higgins et al., 2024).

Within this synthesis, qualitative and mixed-methods evidence was used to explain mechanisms, cue interpretations, and contextual variation, whereas direction-of-effect summaries were restricted to studies reporting extractable quantitative comparisons. Reporting of the synthesis was guided by the Synthesis Without Meta-analysis (SWiM) guideline, with explicit grouping rules (provenance vs. disclosure), consistent mapping of outcomes (credibility vs. trust and message/source/outlet targets), and transparent documentation of how evidence was summarised across heterogeneous designs (Campbell et al., 2020; Popay et al., 2006). Synthesis was conducted on the included full-text set only (Figure 1); reports not retrieved are documented in Appendix B but were not included in study-level extraction or synthesis to protect reliability.

Synthesis was conducted in three linked stages aligned with the research questions and with the exposure constructs reflected in the search strategy:

First stage: Descriptive mapping of the evidence base. The included corpus was characterised in journalism-relevant terms, including publication trends over time, study designs and recruitment approaches, stimulus modality (e.g., text, audiovisual, or image-based news contexts), topic domain (e.g., routine/service news, politics, health/science), and whether studies examined provenance/authorship, disclosure cues, or combined factorial designs crossing provenance and disclosure.

Second stage: Evidence synthesis by manipulation type (provenance vs. disclosure). Findings were synthesised in two primary groups to preserve the conceptual distinction central to the review: Provenance/authorship studies were synthesised around contrasts between AI-authored/AI-assisted news (including automated/algorithmic/robot journalism and generative AI/ChatGPT-described production) and journalist-authored news, noting whether the manipulation implied content differences or attribution-only differences. Disclosure-cue studies were synthesised around contrasts between an explicit AI cue (labels, AI bylines, and/or disclosure/disclaimer statements) and no-disclosure or human-origin cue conditions, where content was otherwise comparable.

Factorial designs crossing provenance and disclosure were synthesised separately to assess whether disclosure effects depend on underlying authorship and to identify provenance × disclosure interactions when reported. Concretely, studies were treated as provenance-focused when the central comparison concerned AI versus human authorship/production, as disclosure-focused when the key manipulation was an explicit AI label/byline/disclaimer attached to otherwise comparable content, and as combined designs when both provenance and disclosure were manipulated simultaneously; in the latter case, they informed disclosure conclusions only when an incremental disclosure contrast could be isolated.

To avoid double-counting, each publication contributed once to direction-of-effect summaries. Where a paper reported multiple studies or outcomes, coding reflected the overall pattern across the relevant contrasts (including “mixed/conditional” where appropriate).

Third stage: Outcome-target synthesis and moderator mapping. Within each synthesis group, results were summarised separately by outcome domain (credibility vs. trust) and, where possible, by target of evaluation (message/article-level vs. journalist/source-level vs. outlet/newsroom-level). Where studies reported multiple indicators within a domain (e.g., accuracy and bias as credibility-related indicators), within-study patterns were described rather than collapsed into a single pooled estimate. Where magnitude information was available (e.g., means/SDs, regression coefficients, confidence intervals), it was reported alongside direction-of-effect summaries to reduce overreliance on statistical significance.

Direction-of-effect coding was used as a descriptive mapping device within broad conceptual groupings, not as evidence that all included studies estimate a single common effect. Because the corpus varies substantially in constructs, targets of evaluation, stimuli, and study design, the synthesis prioritises pattern identification and contextual interpretation over direct cross-study effect comparison. Qualitative and mixed-methods studies were therefore not treated as lower-value evidence; rather, they were used to identify mechanisms, interpretive frames, and contextual conditions that help explain why trust and credibility judgments vary across topics, outlets, and audience settings.

Moderator synthesis was structured around theoretically and practically relevant variations in how AI provenance and disclosure are communicated in news contexts. For disclosure studies, moderators included cue type (label, AI byline, disclosure/disclaimer statement), wording (e.g., “AI-generated” vs. “AI-assisted”), framing (neutral informational vs. warning-like language), placement/prominence (e.g., headline tag/byline/footer), timing, and whether disclosures communicated human oversight. For provenance studies, moderators included topic sensitivity/stakes, modality, and whether AI was framed as autonomous generation versus assistance. Audience-level moderators such as baseline trust in news, AI familiarity, political orientations, and media literacy were synthesised where reported.

Finally, interpretation was informed by the methodological appraisal by treating patterns driven primarily by weaker designs or incomplete reporting more cautiously and giving greater interpretive emphasis to evidence with clearer manipulations, stronger internal validity, and more transparent reporting.

## 3 Results

### 3.1 Included study characteristics

The final synthesis comprised 47 empirical studies with retrievable full-text PDFs (2018–2026), with publication volume increasing markedly after 2023.

Study designs were heterogeneous but were dominated by experiments (*N =* 16) and surveys (*N =* 10); qualitative designs were rare (*N =* 3), and a further subset used mixed or non-standard designs that could not be cleanly classified (*N =* 18). Across studies that reported participant counts, sample sizes ranged widely (median *N* ≈ 336; max≈2,159) and recruitment relied primarily on online panels, with smaller subsets using student/convenience or journalist/professional samples.

Stimuli were predominantly text-based news and commonly covered politics or multiple/general topics; reporting of stimulus modality and other methodological details was often incomplete. Credibility was measured more frequently than trust, and provenance cues were far more common than disclosure-only manipulations, limiting the strength of evidence for RQ2a-RQ2b. Country/region was recorded when reported, but geographical and cultural context was unevenly specified and was rarely analysed directly as a moderator. Accordingly, the current evidence base does not support strong cross-national conclusions about audience trust or credibility responses to AI provenance or disclosure in news. Full study-level characteristics and coding decisions are provided in Appendices A, D.

### 3.2 Quality appraisal overview

Quality appraisal was used to contextualise confidence in the evidence rather than to exclude studies. Appendix E provides study-level notes from the MMAT-based appraisal; here, recurring strengths and limitations are summarised to support cautious interpretation across RQ1-RQ3.

Across the full-text set, 9 of 47 studies (19.1%) reported preregistration (or an equivalent pre-analysis plan), and experimental studies often reported standard safeguards (e.g., attention checks and manipulation checks). However, reporting of cue wording/placement and key design features (e.g., assignment procedures, stimulus provenance, and measurement properties) was frequently incomplete, which limits cross-study comparability and can weaken construct validity.

*Construct validity of AI cues*: Many studies operationalised “AI authorship” via bylines/labels (“assumed AI”) even when the underlying text was held constant, meaning effects may reflect reactions to the cue rather than differences in content quality.

*Confounding between provenance and disclosure*: Several papers bundled authorship attribution, disclosure language, and oversight information, which makes it difficult to isolate the incremental effect of disclosure design.

*External validity*: Samples relied heavily on online panels and convenience recruitment, and most stimuli were single-article vignettes; results may not generalise to repeated exposure, real-world news diets, or high-stakes breaking news contexts.

*Outcome heterogeneity*: Credibility and trust were measured with varied instruments (single items vs. multi-item scales; message vs. source vs. outlet targets), contributing to mixed findings and limiting quantitative pooling.

### 3.3 RQ1: effects of AI provenance/authorship on credibility and trust

RQ1 examined whether attributing news content to AI (vs. a human journalist) affects perceived credibility and trust. Because the included studies vary substantially in design, outcome operationalisation, and reporting practices, a formal meta-analysis was not feasible. Instead, findings are synthesised using narrative synthesis supplemented with structured direction-of-effect coding (positive, negative, no effect, or mixed/conditional). Not all included papers reported extractable quantitative comparisons for each outcome domain.

**Figure 2:**
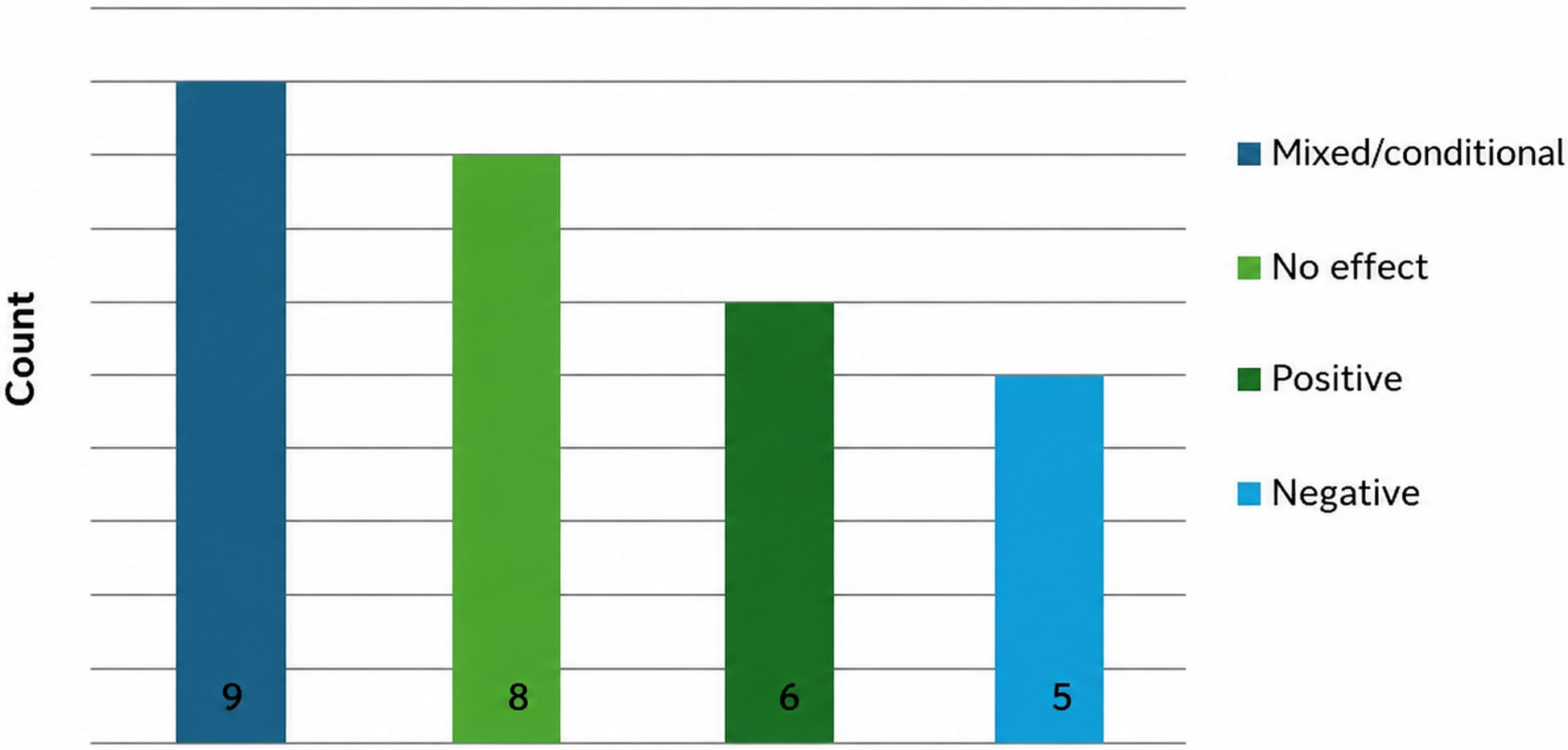
RQ1 (provenance/authorship): direction of effects on credibility (extractable results).

*Credibility.* Of the 47 included studies, 28 reported an extractable direction of effect for AI provenance/authorship on credibility (Figure 2). Across these studies, findings were predominantly null or conditional, with both positive and negative effects observed in smaller subsets. Taken descriptively, the extractable credibility evidence does not consistently indicate a single uniform “AI penalty”; instead, reported findings vary by topic, cue operationalisation, target of evaluation, and study design.

**Figure 3:**
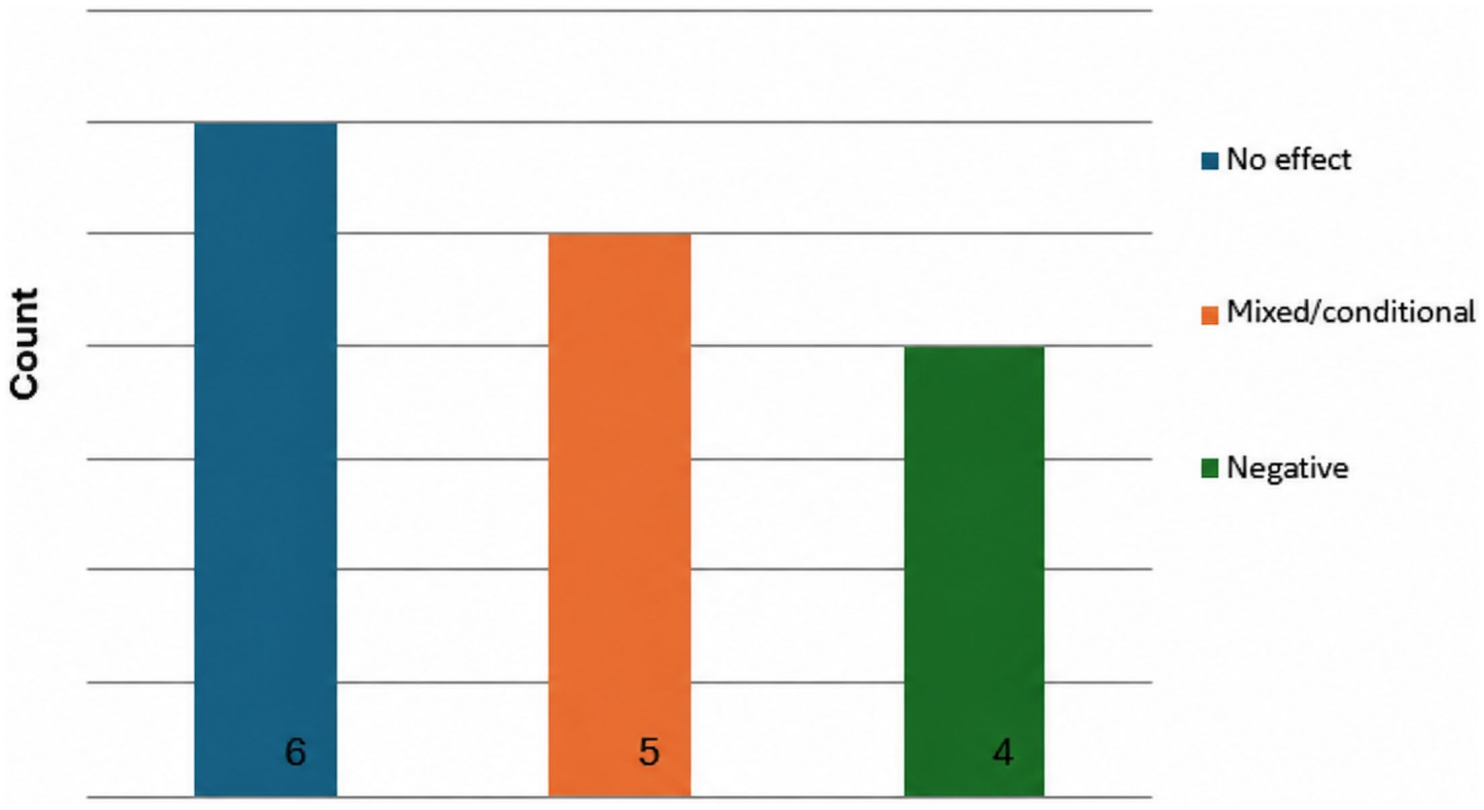
RQ1 (provenance/authorship): direction of effects on trust (extractable results).

*Trust*: Trust outcomes were reported less frequently: only 15 studies provided an extractable direction of effect for AI provenance/authorship on trust (Figure 3). Most findings were null or conditional, while several studies reported lower trust under AI authorship; no clear positive trust effects were observed. This pattern is consistent with the possibility that trust is often operationalised at broader levels (e.g., outlet-level trust) and may be less sensitive to simple byline-based provenance manipulations.

Integrating quantitative and qualitative evidence, a recurring theme is ambivalence: audiences often recognise potential benefits of AI (e.g., speed, scalability, perceived objectivity in routine reporting) while simultaneously expressing concerns about transparency, accountability, and the erosion of journalistic norms. The net effect of AI provenance cues therefore depends on when, where, and how AI involvement is signalled, and what audiences infer from that signal.

Examples from the included studies illustrate why a uniform “AI penalty” is not supported. Several experiments and surveys reported no statistically meaningful difference in credibility or trust when news was attributed to AI versus human authorship (e.g., Jia, 2020; Lermann Henestrosa et al., 2023; Wischnewski and Krämer, 2024).

Where negative effects were observed, they often reflected reduced evaluations of the journalist/source (or headline) when AI involvement was salient (e.g., Graefe et al., 2018; Haim and Maurus, 2023; Gherheș et al., 2025). By contrast, positive credibility effects were rarer and tended to be domain- or context-dependent, such as in routine reporting contexts where automation can be perceived as objective or efficient (e.g., Wölker and Powell, 2021; Sun et al., 2024).

Conditional effects were common in studies that explicitly tested moderators: for example, credibility differences varied by news genre/outlet cues (Liu and Wei, 2019), by issue attitudes and hostile-media perceptions (Jia and Liu, 2021; Cloudy et al., 2023), and by respondents’ social trust in AI/human information production (Liu et al., 2026).

### 3.4 RQ2a: main effects of AI disclosure cues on credibility and trust

RQ2a focused on the main incremental effect of disclosure cues-labels, AI bylines, and disclosure/disclaimer statements-intended to inform audiences that AI was involved in producing or modifying news content. In the included full-text set, disclosure manipulations were uncommon and were often embedded in factorial designs alongside provenance/authorship cues, which limited the number of directly comparable disclosure effects. Accordingly, studies that manipulated both authorship/provenance and disclosure were treated as combined designs, rather than as straightforward disclosure-only tests, unless the study reported a separable incremental disclosure effect. Table 3 summarises the disclosure-cue evidence base (*N =* 10) and indicates which studies provide extractable disclosure-only contrasts.

**Table 3:** Disclosure cue evidence base (RQ2a-RQ2b): cue operationalisations and extractable disclosure effects (*N =* 10 studies).

Study (evidence type)	Disclosure cue and comparator (summary)	Disclosure effect on credibility	Disclosure effect on trust
Blom et al. (2025) (qualitative interviews)	Labeling of generative AI discussed using labeled and unlabeled examples; no standardised experimental disclosure manipulation.	NR	NR
Forstner et al. (2025) (experiment; simulated recommender)	Image trust labels (Verified, AI-generated, Edited) with clickable details; compared with a no-label baseline.	NR	No effect
Sadri et al. (2025) (factorial experiment)	AI byline describing an AI language model (shown near top and repeated); compared with a human byline and crossed with authorship source (AP vs. ChatGPT vs. Gemini).	NR	NR
Rossner et al. (2024) (survey experiment)	Origin label at top of article (Human vs. AI) with three disclosure conditions: no label, correct label, and swapped/misleading label.	No effect	No effect
Zoizner et al. (2025) (conjoint experiment)	Participants informed items may be AI-generated; authorship attribute (human, AI, none) and a fictitious AI-only outlet varied alongside other cues (disclosure not isolated as a single treatment).	N/A	N/A
Spinde et al. (2025) (experiments; media-literacy intervention)	Bias labels/annotations attributed to Human vs. AI (teaching phase); labels removed in testing phase; compared with control and other visualisation combinations.	Mixed or conditional	NR
DeVerna et al. (2024) (preregistered experiment)	Fact-checking aid source and visibility varied: control, LLM optional, LLM forced, and human fact-check conditions.	Positive	NR
Sun et al. (2024) (survey)	Public perceptions of AI in journalism; no disclosure or label manipulation of specific news stimuli.	N/A	N/A
Lermann Henestrosa and Kimmerle (2024) (preregistered experiment)	Identical GPT-generated text labeled as AI-authored vs. human-authored (assumed authorship cue).	No effect	NR
Fernández-Barrero and Serrano-Martín (2025) (mixed methods; newsroom transparency)	Reported disclosure practices and perceived trust implications; descriptive (no experimental disclosure manipulation).	Mixed or conditional	Mixed or conditional

NR, not reported or not extractable as an incremental disclosure effect; N/A, disclosure not evaluated as an independent treatment.

Credibility. Evidence on disclosure cues was limited. Only five studies provided an extractable disclosure effect on credibility. Across these studies, findings were predominantly null or conditional (two no-effect findings, two mixed/conditional findings), with one study reporting a positive effect; no study provided an unambiguous negative main effect attributable solely to disclosure cues.

Trust. Trust outcomes for disclosure cues were even rarer (three studies). Two reported no effect and one reported mixed/conditional effects; no study provided an unambiguous positive or negative main effect of disclosure on trust.

Taken together, the available full-text evidence does not support treating AI disclosure as a uniform “warning label” that consistently reduces credibility or trust. The extractable evidence instead suggests that disclosure main effects are usually null or conditional. The scarcity of disclosure-only manipulations remains a key empirical gap.

### 3.5 RQ2b: do cue characteristics condition disclosure effects?

RQ2b focused on whether cue characteristics condition disclosure effects. Evidence bearing on cue design is more limited than evidence on disclosure as a main effect, but the available studies suggest that audience responses depend less on disclosure per se than on what the cue implies about automation and accountability. For example, some label formats were interpreted as useful verification signals and increased perceived trustworthiness in interface evaluations (Forstner et al., 2025), whereas other disclosure implementations produced little to no change in credibility (Rossner et al., 2024; Lermann Henestrosa and Kimmerle, 2024).

Importantly, disclosure research in this domain is often embedded in factorial designs or bundled with authorship attributions, which can obscure the incremental effect of disclosure wording and placement. Qualitative studies indicate that audiences generally support transparency but differ in how they interpret “AI-generated” versus “AI-assisted” cues and when they expect disclosure (Blom et al., 2025; Strikovic and Cools, 2025).

Evidence of potential backlash is also present: in a conjoint setting, AI attribution/disclosure cues were associated with mistrust and reduced willingness to engage, rather than improved openness to cross-cutting news (Zoizner et al., 2025). Overall, the current evidence base supports treating disclosure as a design problem (specific cue, wording, placement, and oversight framing) rather than a single uniform treatment, but it does not yet isolate any one cue feature as consistently beneficial across contexts. More specifically, the current evidence suggests that disclosure should be treated as a design problem because the included studies do not manipulate a single, equivalent “AI disclosure” treatment. Instead, they vary substantially in cue type, wording, placement, informational richness, and whether disclosure is bundled with other source cues. For example, Rossner et al. (2024) varied a top-of-article origin label across no-label, correct-label, and misleading-label conditions and found no clear credibility or trust effect, while Lermann Henestrosa and Kimmerle (2024) similarly reported no credibility difference when identical GPT-generated text was labeled as AI-authored rather than human-authored. By contrast, Spinde et al. (2025) found mixed or conditional effects in a media-literacy setting using human-versus-AI bias labels, and DeVerna et al. (2024) reported a positive credibility effect when source and visibility cues were tied to fact-checking support rather than merely signaling automation. Other studies further show why design matters conceptually: Forstner et al. (2025) used image trust labels with clickable details, suggesting that explanatory depth and interface integration shape how such cues are interpreted, while qualitative work by Blom et al. (2025) and Strikovic and Cools (2025) indicates that audiences distinguish between “AI-generated” and “AI-assisted” wording and care about when and how disclosure is presented. Finally, in bundled or conjoint designs, such as Zoizner et al. (2025), AI attribution/disclosure cues could coincide with reduced willingness to engage, but because authorship, source, and outlet cues varied simultaneously, these studies do not isolate disclosure as a single treatment. Taken together, these examples show that disclosure effects depend on what the cue communicates about automation, oversight, verification, and accountability; this is why the literature currently supports a design-sensitive interpretation rather than a uniform disclosure effect.

### 3.6 RQ3: when and why do these effects vary?

RQ3 examined moderators and contextual conditions that shape when AI provenance and/or disclosure cues influence audience judgments. Only 9 of the 47 included full-text studies explicitly tested moderators in their analyses, which limits strong inferences about boundary conditions. Here, boundary conditions refer to the circumstances under which AI provenance or disclosure cues are more likely to matter rather than yield uniform effects, such as topic and genre, political salience, partisan identity or prior issue attitudes, hostile-media perceptions, outlet/source context, and audience familiarity with AI. Where moderators were tested, they most often involved political identity or issue attitudes, prior knowledge/awareness of automated journalism or AI, and contextual features such as outlet reputation, source cues, or stimulus valence. Across the moderator-focused studies, several patterns recur at a conceptual level. First, politically salient contexts appear more likely to produce conditional effects. Here, politically salient settings refer to news contexts in which the topic, source, or framing is tied to contested political identities, partisan commitments, or strongly held prior issue attitudes, making audiences more likely to interpret AI attribution through those commitments rather than as a neutral authorship cue. This includes polarising topics, partisan source contexts, and situations in which hostile-media perceptions are likely to be activated. Second, audience familiarity with AI and awareness of automated journalism can shape how provenance or disclosure cues are interpreted: for some audiences, AI cues may signal competence (e.g., efficiency or consistency), whereas for others they may trigger norm-based concerns (e.g., lack of accountability, weakened human judgment, or diminished editorial responsibility). Third, organisational context matters: the same AI cue may be interpreted differently depending on the baseline credibility of the news source and the perceived norms of the content genre (e.g., routine/service reporting versus interpretive or high-stakes reporting).

Where moderators were tested, topic and genre were recurring boundary conditions. For instance, effects of algorithmic authorship varied across finance versus sports contexts in experimental work (Wölker and Powell, 2021), and machine-authorship effects differed by news genre and organisational cues in a genre-sensitive design (Liu and Wei, 2019).

One important set of boundary conditions involved politically salient settings. When topics were politically polarising, when audiences held strong partisan identities or prior issue attitudes, or when hostile-media perceptions were activated, AI attribution was less likely to function as a neutral cue and more likely to be interpreted through prior political commitments. Politically salient settings were therefore more likely to yield conditional effects. Studies reported interactions (or theoretically motivated tests) involving political identity, issue attitudes, or hostile-media perceptions when AI involvement was made salient (Jia and Liu, 2021; Cloudy et al., 2023; Wischnewski and Krämer, 2024). In conjoint evidence, AI attribution did not operate as a “neutral alternative” to partisan sources and could instead trigger resistance (Zoizner et al., 2025). Audience knowledge and familiarity with automated journalism/AI shaped interpretation of cues. Knowledge of automated journalism moderated evaluations of algorithmically generated news (Jang et al., 2024). Survey evidence further suggests that expectations about AI’s role in journalism co-vary with both trust-related and objectivity-related attitudes: trust-related issues concern whether AI-involved journalism is seen as reliable, accountable, and worthy of reliance, whereas objectivity-related issues concern whether AI is perceived as more neutral, consistent, and less biased than human journalists, or instead as lacking contextual editorial judgment (Sun et al., 2024; Yeste-Piquer et al., 2025). These attitudes did not vary in a single direction; AI could be viewed more positively when associated with efficiency, consistency, or reduced human bias, but more negatively when audiences prioritised responsibility, transparency, and human oversight.

Finally, studies that examine broader dispositions suggest that social trust can condition credibility judgments across AI, human, and hybrid authorship categories (Liu et al., 2026). Disclosure effects also hinge on comprehension and what the cue implies about oversight: interface work shows that label design affects perceived usefulness/trust but may be misunderstood (Forstner et al., 2025), and experimental work suggests that evaluative information and oversight framing can shift interpretations of AI involvement (Lermann Henestrosa et al., 2023).

Geographical and cultural setting is a plausible but under-examined moderator. Country/region was recorded when reported during extraction, but studies varied in how fully they described national, cultural, political, or media-system context, and few directly compared countries or tested cultural setting as a moderator. The current evidence therefore does not support strong claims that audiences in one geographical context systematically trust AI-involved journalism more or less than audiences in another; rather, geography is best understood as a likely source of heterogeneity that remains insufficiently tested.

These apparent inconsistencies reflect not only uneven reporting in the primary studies but also the breadth of the present review. Because the corpus spans different constructs, targets of evaluation, stimuli, and designs, the findings should not be read as if all studies were estimating the same underlying treatment effect on a common scale. Instead, the direction-of-effect summaries are used descriptively to map broad patterns within conceptual groupings, while qualitative and mixed-methods studies are used to explain how audience responses vary by topic, genre, outlet context, and expectations of oversight and accountability. In this sense, qualitative studies do not count for less because they do not yield an extractable effect size; they contribute differently by clarifying mechanisms, interpretive frames, and the conditions under which AI cues are accepted, resisted, or understood ambivalently. Several fields were nevertheless frequently coded as “Unclear” due to limited methodological detail in the original reports, especially for stimulus modality, cue placement/prominence, design classification, and measurement properties, which constrains finer-grained comparison.

## 4 Discussion

This review synthesised the accessible full-text evidence on how AI involvement in news production (provenance/authorship) and explicit disclosure cues (labels, bylines, disclosure/disclaimer statements) shape audience perceptions of credibility and trust. Restricting synthesis to the 47 studies for which a complete full-text PDF was retrievable and could be coded using a common extraction template prioritised reliability and comparability of the dataset. However, this approach also highlights a key structural imbalance in the evidence base: most included studies examine provenance/authorship cues (*N =* 36), while explicit tests of disclosure cues are comparatively uncommon (*N =* 10 in total; only one in a disclosure-only design). Overall, audience responses to AI-involved journalism are best characterised as contingent, varying by design, context, and measurement target, rather than uniformly positive or negative.

### 4.1 Interpreting the findings across RQ1-RQ3

Across RQ1, AI provenance/authorship cues produced heterogeneous outcomes. For credibility, the most common pattern was mixed/conditional effects (9 of 28 extractable contrasts), followed by no effect (8), with fewer clearly positive (6) or negative (5) directions. For trust, extractable findings were more likely to indicate no effect (6 of 15) or mixed/conditional effects (5), with the remainder indicating a negative direction (4). Two implications follow. First, audiences do not consistently discount AI-involved journalism on article credibility grounds. Second, trust-related judgements appear more consistently non-positive than credibility outcomes: within the extractable evidence, no study showed a clearly positive trust effect of AI authorship. This pattern is consistent with the possibility that AI cues operate less as a purely epistemic signal (“this information is wrong”) and more as a normative or relational cue (“this is not the type of actor/process I want responsible for journalism”), particularly when the evaluation target is the author or newsroom rather than the informational content alone.

These summaries should therefore not be interpreted as quasi-meta-analytic estimates or as evidence of a generalised “no effect.” Rather, they provide a descriptive map of how often extractable studies reported positive, negative, null, or conditional findings within broad conceptual categories. The review supports heterogeneity and context dependence more confidently than any single pooled conclusion, particularly because the trust evidence base is comparatively small and because qualitative work shows that responses vary with topic, outlet context, cue framing, and perceived accountability.

RQ2a (main disclosure effects) should be interpreted with particular caution because the extractable evidence base is small (credibility: 5 studies; trust: 3 studies), and disclosure is often examined only in combination with provenance cues. Within the limited extractable set, disclosure cues most often produced no effect or conditional effects, rather than a uniform reduction in perceived credibility or trust. Importantly, this does not imply that disclosure never matters; rather, it indicates that, within the retrievable full-text evidence base, rigorous, directly comparable tests of disclosure as a main effect remain scarce. Given the prominence of transparency in normative debates about AI and journalism, the limited volume of disclosure-only evidence is itself one of the review’s key findings.

RQ2b (cue-characteristic contingencies) is even more limited. The available literature suggests that wording, framing, placement/prominence, and stated human oversight may condition reactions to disclosure, but these features are often bundled with provenance or accountability information. As a result, the literature currently supports the claim that disclosure effects are design-sensitive, but not strong prescriptions about which specific disclosure formulations reliably mitigate scepticism or reinforce confidence across contexts.

Geographical and cultural setting is another plausible source of heterogeneity, but it remains under-examined. Although country/region was captured during extraction when reported, few studies directly compare national contexts or test media-system differences. The review therefore cannot conclude that geographical context significantly alters the overall pattern in a systematic way; instead, it identifies cross-cultural variation as a credible possibility that requires targeted comparative research.

More broadly, RQ3 emphasised the field’s reliance on theorised (rather than consistently tested) contingencies. Only 9 of the 47 studies explicitly tested moderators. As a result, many plausible boundary conditions, such as label wording (e.g., “AI-generated” vs. “AI-assisted”), prominence and placement of cues, and whether disclosures communicate human oversight, remain more often proposed than systematically evaluated. A practical consequence is that the literature provides more confidence that effects are heterogeneous than it does about which specific disclosure designs reliably mitigate scepticism or reinforce confidence across contexts.

### 4.2 Conceptual implications for trust, credibility, and AI cues in journalism

The review reinforces the analytical value of separating provenance/authorship cues from disclosure cues. Provenance information communicates who (or what) produced the content and can trigger inferences about competence, objectivity, bias, and the appropriateness of delegating journalistic work to machines. Disclosure cues, by contrast, are communicative signals about transparency that may shift attention from the message to the production process and raise questions of accountability and oversight. This distinction is consistent with broader work on online credibility judgement, which shows that audiences often rely on cues about source and production context when evaluating information quality and trustworthiness (Flanagin and Metzger, 2007; Metzger and Flanagin, 2013), and with research on heuristic reactions to algorithmic or machine agents (Dietvorst et al., 2015; Sundar and Kim, 2019). Conflating provenance and disclosure risks misattributing effects to “AI production” when they are driven by transparency framing, or vice versa, and makes it harder to explain why some studies find minimal differences at the message level while still observing normative discomfort at the source level.

**Figure 4:**
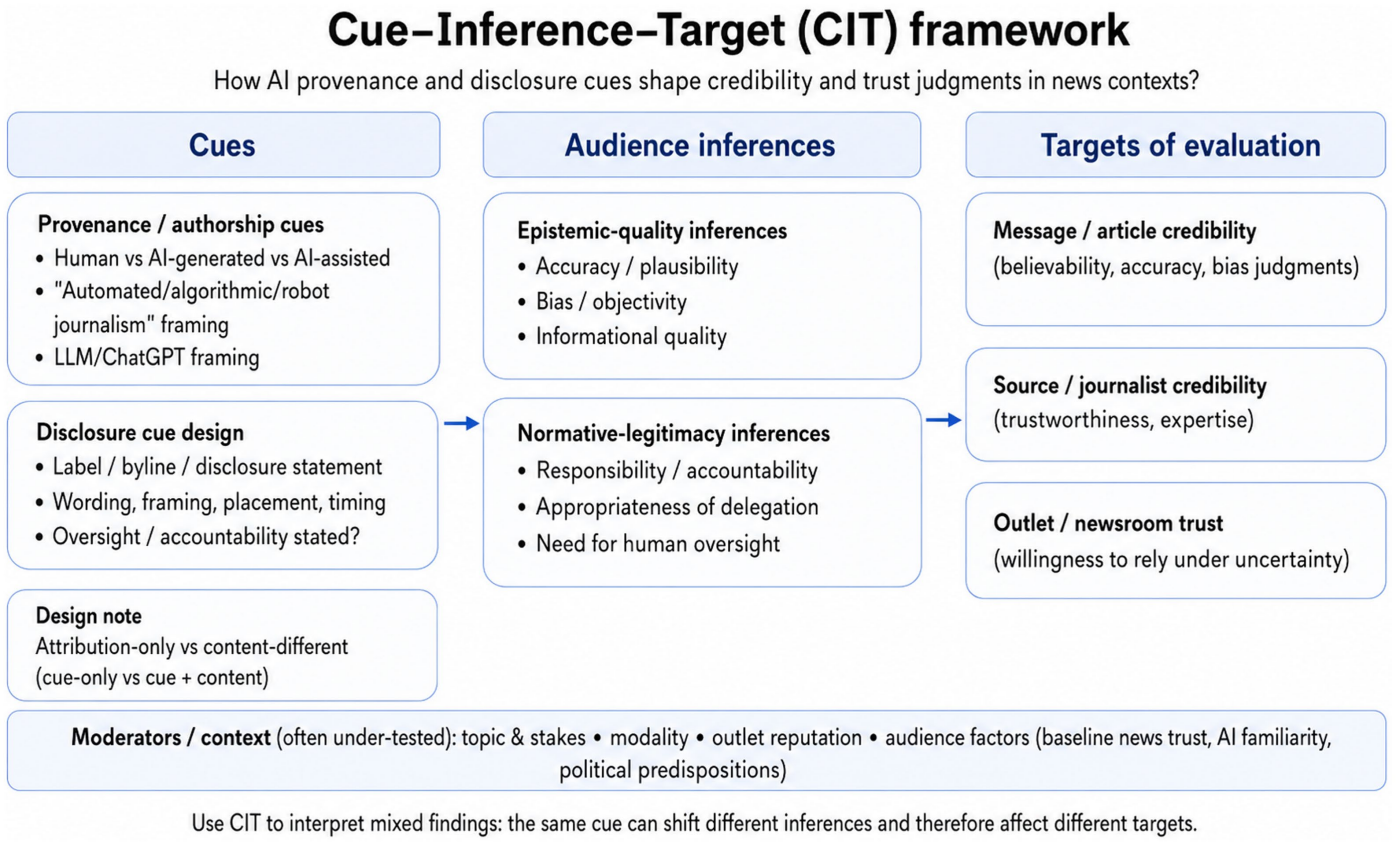
Cue–inference–target (CIT) framework for AI provenance and disclosure effects on trust and credibility.

To organise these heterogeneous findings, Figure 4 presents a Cue–Inference–Target (CIT) framework. The framework treats provenance and disclosure as distinct cue types and positions them as inputs that shape the inferences audiences draw, which in turn influence evaluations at different targets. In addition to the cue itself, the CIT framework highlights whether studies compare attribution-only conditions (identical content with different provenance/disclosure information) or content-different conditions (AI and human versions differ), because these designs test different mechanisms and are not directly comparable. The Cue–Inference–Target framework is proposed as an integrative synthesis framework derived from recurring patterns in the reviewed evidence, rather than as a tested mediation model; direct measurement of the proposed inference pathways remains limited in the current literature.

Within the C-I-T framework, AI-related cues are expected to operate through at least two partially independent inference pathways. One pathway involves epistemic-quality inferences: judgements about accuracy, plausibility, bias/objectivity, and perceived informational quality. The other involves normative-legitimacy inferences: judgements about responsibility, accountability, and whether AI involvement fits expectations about how journalism should be produced. These pathways help explain a recurring pattern in the included evidence: message/article credibility can remain stable even when source- or outlet-level trust is more contested, because scepticism may be directed at the legitimacy of the production process rather than at the factuality of a specific item.

The framework also clarifies why construct specification and outcome targeting are central for comparability. Trust is commonly conceptualised as a willingness to rely on an actor or institution under uncertainty and vulnerability (Mayer et al., 1995) and, in news contexts, often reflects confidence in journalistic processes such as accuracy and assessment (Kohring and Matthes, 2007). Credibility measures more often capture perceived believability and informational quality at the message level (Appelman and Sundar, 2016; Metzger and Flanagin, 2013). More specifically, the reviewed evidence suggests that audience acceptance of AI involvement depends not only on whether AI use is disclosed, but also on whether the disclosure clarifies the role of AI, the presence of human review, and where editorial responsibility remains. Qualitative studies indicate that audiences generally support transparency but distinguish between “AI-generated” and “AI-assisted” cues and care about when and how disclosure is provided (Blom et al., 2025; Strikovic and Cools, 2025). Related interface and experimental evidence further suggests that label design, explanatory detail, and oversight framing shape how such cues are interpreted (Forstner et al., 2025; Lermann Henestrosa et al., 2023). Taken together, these studies suggest, cautiously, that visible human oversight and clear editorial accountability can moderate normative-legitimacy judgments about AI involvement in journalism. For news organisations, the evidence suggests three practice-relevant implications.

First, the credibility implications of AI use appear context-dependent rather than uniformly negative. Routine or clearly data-driven formats may be less vulnerable to credibility penalties than politically salient or high-stakes contexts, where audiences may be more attentive to authorship cues and interpret AI use through the lens of institutional responsibility and legitimacy.

Second, transparency should be treated as a design and communication problem, not a binary decision to disclose or not disclose. Although the disclosure evidence base in the included full-text set is limited, it does not support the claim that disclosure inevitably harms trust. Instead, the available evidence and the qualitative themes suggest that how AI involvement is communicated matters: disclosures that specify the role of AI (e.g., summarising, translation, drafting), clarify the presence of human editorial review, and reaffirm organisational accountability may better match audience expectations than generic labels that simply announce “AI-generated” without context.

Third, newsroom decision-makers should anticipate the possibility that audiences evaluate trust at the institutional level even when message credibility appears unaffected. This distinction matters for reputational risk: an article may be perceived as plausible or accurate while still triggering broader concerns about legitimacy, professional standards, and accountability. Communicating editorial safeguards, verification procedures, correction policies, and boundaries of automation, may therefore matter as much as communicating the mere fact of AI involvement.

### 4.3 Limitations

This review has several limitations that should temper interpretation.

Full-text availability constraint. The PRISMA-tracked master list includes additional records, but synthesis was intentionally restricted to studies for which full texts could be retrieved through institutional subscriptions and open-access channels and coded comparably. Direct author-contact retrieval was not systematically pursued, which may have increased the number of reports not retrieved. This decision prioritises reliability and auditability of the extracted dataset, but it may introduce availability bias if retrievable articles differ systematically from non-retrieved or access-restricted reports (e.g., by outlet type, geography, or reporting norms). Consequently, results should be interpreted as characterising the accessible full-text evidence base rather than the entire universe of potentially relevant studies.

Reviewer process. Screening and coding decisions were conducted by the first author. Although a prespecified protocol, codebook, structured logs, and consistency checks were used, the absence of independent duplicate screening and extraction may increase the risk of selection or coding error.

Heterogeneity and reporting limitations. Included studies varied substantially in design, topic, modality, operationalisation of AI involvement, and measurement of trust/credibility. Effect sizes were inconsistently reported and rarely comparable, limiting the feasibility of quantitative pooling. As a result, synthesis relied on structured narrative integration and direction-of-effect coding, which does not capture magnitude and can understate nuance. Moreover, disclosure cue designs (wording, placement, oversight information) were often under-described, limiting cross-study inferences about “what works” in transparency.

A further limitation is that the breadth of the review required comparison across conceptually related but not fully equivalent studies. Although the narrative synthesis and outcome-target mapping were designed to reduce inappropriate aggregation, the review necessarily trades depth within narrower contexts for breadth across the field; accordingly, direction-of-effect summaries should be read as descriptive evidence maps rather than as estimates of a single underlying effect.

Scope constraints. Searches were limited to Scopus and Web of Science, English-language publications, and the prespecified search strings. Evidence outside these databases, in other languages, or in grey literature may therefore be missing. Finally, the evidence base is temporally skewed toward recent years, and conclusions may evolve as newsroom practices, disclosure norms, and audience expectations change. In addition, geographical and media-system representation was unevenly reported, and few studies directly compared cultural contexts; combined with the English-language search restriction, this limits strong cross-cultural generalisation.

### 4.4 Future research directions

Three directions follow directly from the gaps identified.

Build a disclosure-design evidence base with clean causal designs. The most urgent empirical gap is rigorous testing of disclosure cue design: wording (“AI-generated” vs. “AI-assisted”), framing (neutral vs. warning-like), placement/prominence (headline tag vs. byline vs. footer), timing, and explicit statements of human oversight/accountability. Critically, future work should prioritise factorial designs that disentangle provenance from disclosure (e.g., 2 × 2: provenance cue per disclosure presence/format) and explicitly state whether stimuli are attribution-only or content-different. This would allow clearer causal attribution and more actionable guidance for newsroom transparency policies.

Standardise measurement and target specification. Future studies should distinguish message credibility from source/outlet trust, report scale properties and reliability, and where feasible, report effect sizes in reusable formats. Multi-level outcome designs (article, author, outlet) are especially valuable for identifying whether AI cues primarily affect perceived informational quality, perceived legitimacy, or both. A practical “minimum reporting set” for this literature would include: the exact cue wording, cue placement, whether oversight is stated, target of evaluation, and at least one validated trust/credibility measure.

Expand moderator testing and move beyond single-shot designs.

Moderator evidence remains limited. Future work should systematically test how baseline news trust, AI familiarity/AI literacy, political predispositions, topic sensitivity/stakes, and outlet reputation condition responses to AI provenance and disclosure. Cross-national and cross-media-system designs, alongside longitudinal research, are particularly important because public “folk theories” about AI and journalism likely vary across media systems and may change as generative AI becomes more familiar and newsroom disclosure norms stabilise.

## 5 Conclusion

Overall, within the accessible full-text evidence base, the extractable provenance/authorship studies do not consistently indicate a uniform “AI penalty.” Among the studies from which a direction of effect could be extracted, credibility findings were predominantly null or conditional, while trust findings were fewer and more often non-positive than positive. These patterns should therefore be read as evidence of heterogeneity and context dependence, rather than as proof that AI involvement carries no penalty. Instead, audience responses appear to vary with contextual and design factors, including topic, baseline trust, and whether accountability or human oversight is signalled. The target of evaluation also matters: message-level credibility may remain relatively stable even when trust in the producing actor, outlet, or production process is more contested. Accordingly, the Cue–Inference–Target (CIT) framework is proposed as a synthesis framework for organising these recurring patterns, rather than as a definitive claim that AI involvement has no credibility or trust costs.

Evidence on disclosure cues remains comparatively limited and is dominated by combined (provenance and disclosure) designs; therefore, strong universal prescriptions about disclosure implementation cannot yet be made with confidence. Nevertheless, the available findings suggest that disclosure is unlikely to function as a uniform “warning label.” Instead, disclosure appears to operate as an interpretive frame whose effects depend on wording, placement, and whether disclosures clarify the *role* of AI and make accountability explicit. For journalism practice, transparency is therefore best treated as part of a broader accountability package (e.g., specifying whether AI was used for drafting, translation, summarisation, or image generation; indicating editorial review; and clearly communicating responsibility and correction processes). For research, the priority is the use of preregistered factorial designs that separate provenance from disclosure, systematically vary disclosure features (verbatim wording, prominence/placement, and modality), and apply validated measures that distinguish message credibility from source/outlet trust. Replication across topics, outlets, and cultural contexts will be essential as AI systems, newsroom practices, and public expectations continue to evolve.

## Data Availability

The original contributions presented in the study are included in the article/Supplementary material, further inquiries can be directed to the corresponding author.

## References

[ref1] Altay S. Gilardi F. (2024). People are skeptical of headlines labeled as AI-generated, even if true or human-made, because they assume full AI automation. PNAS nexus 3:pgae403. doi: 10.1093/pnasnexus/pgae403, 39359399 PMC11443540

[ref2] Appelman A. Sundar S. S. (2016). Measuring message credibility: construction and validation of an exclusive scale. J. Mass Commun. Q. 93, 59–79. doi: 10.1177/1077699015606057

[ref3] Barrolleta L. A. L. R. Sandoval-Martín T. (2024). Artificial intelligence versus journalists: the quality of automated news and bias by authorship using a Turing test. Anàlisi: Q. Comun. Cultura 70, 15–36. doi: 10.5565/rev/analisi.3681

[ref4] Bien-Aimé S. Wu M. Appelman A. Jia H. (2025). Who wrote it? News readers’ sensemaking of AI/human bylines. Commun. Rep. 38, 46–58. doi: 10.1080/08934215.2024.2424553

[ref5] Blom J. N. Heiselberg L. van Dalen A. Andersen I. (2025). I think it’s exciting and frightening at the same time: audience sentiments toward the use and Labeling of generative AI in journalism. J. Mass Commun. Q. doi: 10.1177/10776990251385943

[ref6] Bramer W. M. Giustini D. de Jonge G. B. Holland L. Bekhuis T. (2016). De-duplication of database search results for systematic reviews in EndNote. J. Med. Libr. Assoc. 104, 240–243. doi: 10.3163/1536-5050.104.3.014, 27366130 PMC4915647

[ref7] Bramer W. M. Rethlefsen M. L. Kleijnen J. Franco O. H. (2017). Optimal database combinations for literature searches in systematic reviews: a prospective exploratory study. Syst. Rev. 6:245. doi: 10.1186/s13643-017-0644-y, 29208034 PMC5718002

[ref8] Bykov I. A. Kurushkin S. V. (2025). “Communication strategy of AI-application in news reporting: human values or technological effectiveness?” in 2025 Communication Strategies in Digital Society Seminar (ComSDS) (51–54). IEEE.

[ref9] Campbell M. McKenzie J. E. Sowden A. Katikireddi S. V. Brennan S. E. Ellis S. . (2020). Synthesis without meta-analysis (SWiM) in systematic reviews: reporting guideline. BMJ 368:16890. doi: 10.1136/BMJ.l6890 PMC7190266 31948937

[ref10] Carlson M. (2015). The robotic reporter: automated journalism and the redefinition of labor, compositional forms, and journalistic authority. Digit. Journal. 3, 416–431. doi: 10.1080/21670811.2014.976412

[ref11] Chen M. Koratsky I. C. Liu F. Nah S. (2025). Generative AI in the news: the impact of framing on public attitude and engagement. Int. Conf. Hum. Comput. Int. 22, 3–21. doi: 10.1007/978-3-031-93418-6_1

[ref12] Chesney R. Citron D. (2019). Deepfakes and the new disinformation war: the coming age of post-truth geopolitics. Foreign Aff. 98:147.

[ref13] Clerwall C. (2014). Enter the robot journalist: users' perceptions of automated content. Journal. Pract. 8, 519–531. doi: 10.1080/17512786.2014.883116

[ref14] Cloudy J. Banks J. Bowman N. D. (2023). The str (AI) ght scoop: artificial intelligence cues reduce perceptions of hostile media bias. Digit. Journal. 11, 1577–1596. doi: 10.1080/21670811.2021.1969974

[ref15] Cools H. Diakopoulos N. (2024). Uses of generative AI in the newsroom: mapping journalists’ perceptions of perils and possibilities. Journal. Pract. 20, 878–896. doi: 10.1080/17512786.2024.2394558

[ref16] Danry V. Pataranutaporn P. Groh M. Epstein Z. (2025). “Deceptive explanations by large language models lead people to change their beliefs about misinformation more often than honest explanations,” in *Proceedings of the 2025 CHI Conference on Human Factors in Computing Systems* (1–31).

[ref17] Danzon-Chambaud S. (2021). A systematic review of automated journalism scholarship: guidelines and suggestions for future research. Open Res. Europe 1:4. doi: 10.12688/openreseurope.13096.1, 37645115 PMC10445913

[ref18] DeVerna M. R. Yan H. Y. Yang K. C. Menczer F. (2024). Fact-checking information from large language models can decrease headline discernment. Proc. Natl. Acad. Sci. 121:e2322823121. doi: 10.1073/pnas.2322823121, 39630865 PMC11648662

[ref19] Diakopoulos N. (2019). Automating the news: how algorithms are rewriting the media. Cambridge, MA: Harvard University Press.

[ref20] Dietvorst B. J. Simmons J. P. Massey C. (2015). Algorithm aversion: people erroneously avoid algorithms after seeing them err. J. Exp. Psychol. Gen. 144, 114–126. doi: 10.1037/xge0000033, 25401381

[ref21] Dobber T. Hameleers M. Starke C. van der Meer T. (2025). A Beacon of trustworthiness in a sea of disinformation: does news coverage about the dangers of generative AI cause people to flock to journalism? Int. J. Commun. 19328036:19.

[ref22] Fernández-Barrero M. Á. Serrano-Martín C. (2025). Are the media transparent in their use of AI? Self-regulation and ethical challenges in newsrooms in Spain. J. Media 6:152. doi: 10.3390/journalmedia6030152

[ref23] Flanagin A. J. Metzger M. J. (2007). The role of site features, user attributes, and information verification behaviors on the perceived credibility of web-based information. New Media Soc. 9, 319–342. doi: 10.1177/1461444807075015

[ref24] Forstner S. L. Lysova Y. Starke A. D. Trattner C. (2025). Evaluating Image Trust Labels in a News Recommender System. In Proceedings of the 13th International Workshop on News Recommendation and Analytics (INRA 2025), co-located with the 2025 ACM Conference on Recommender Systems (RecSys 2025), Prague, Czech Republic, September 26, 2025. CEUR Workshop Proceedings, Vol. 4056. Aachen, Germany: CEUR-WS.

[ref25] Gavurova B. Skare M. Hynek N. Moravec V. Polishchuk V. (2024). An information-analytical system for assessing the level of automated news content according to the population structure–a platform for media literacy system development. Technol. Forecast. Soc. Chang. 200:123161. doi: 10.1016/j.techfore.2023.123161

[ref26] Gherheș V. Fărcașiu M. A. Cernicova-Buca M. Coman C. (2025). AI vs. human-authored headlines: evaluating the effectiveness, trust, and linguistic features of ChatGPT-generated clickbait and informative headlines in digital news. Information 16:150. doi: 10.3390/info16020150

[ref27] Gong C. (2023). AI voices reduce cognitive activity? A psychophysiological study of the media effect of AI and human newscasts in Chinese journalism. Front. Psychol. 14:1243078. doi: 10.3389/fpsyg.2023.1243078, 38078218 PMC10701901

[ref28] Govers J. Pareek S. Velloso E. Goncalves J. (2025). Feeds of distrust: investigating how AI-powered news chatbots shape user trust and perceptions. ACM Trans. Interact. Intell. Syst. 15, 1–31. doi: 10.1145/3722227

[ref29] Graefe A. Haim M. Haarmann B. Brosius H.-B. (2018). Readers’ perception of computer-generated news: credibility, expertise, and readability. Journalism 19, 595–610. doi: 10.1177/1464884916641269

[ref30] Gusenbauer M. Haddaway N. R. (2020). Which academic search systems are suitable for systematic reviews or meta-analyses? Evaluating retrieval qualities of Google scholar, PubMed, and 26 other resources. Res. Synth. Methods 11, 181–217. doi: 10.1002/jrsm.1378, 31614060 PMC7079055

[ref31] Haim M. Graefe A. (2017). Automated news: better than expected? Digit. J. 5, 1044–1059. doi: 10.1080/21670811.2017.1345643

[ref32] Haim M. Knöpfle P. Breuer J. (2025). Contextual changes, credible conclusions? A direct and conceptual replication of Shen et al.'s (2019) study on online image credibility. Media Psychol. 22, 1–23. doi: 10.1080/15213269.2025.2595452

[ref33] Haim M. Maurus K. (2023). Stereotypes and sexism? Effects of gender, topic, and user comments on journalists’ credibility. Journalism 24, 1442–1461. doi: 10.1177/14648849211063994

[ref34] Heiselberg L. Blom J. N. van Dalen A. (2022). Automated news reading in the neural age: audience reception and perceived credibility of a news broadcast read by a neural voice. Journal. Stud. 23, 896–914. doi: 10.1080/1461670X.2022.2052346

[ref35] Higgins J. P. T. Thomas J. Chandler J. Cumpston M. Li T. Page M. J. . (2024). Cochrane handbook for systematic reviews of interventions (version 6.5, updated august 2024). Cochrane. Available online at: https://www.cochrane.org/handbook (Accessed February 11, 2026).

[ref36] Hong Q. N. Fàbregues S. Bartlett G. Boardman F. Cargo M. Dagenais P. . (2018). The mixed methods appraisal tool (MMAT) version 2018 for information professionals and researchers. Educ. Inf. 34, 285–291. doi: 10.3233/EFI-180221

[ref37] Jang W. Kwak D. H. Bucy E. (2024). Knowledge of automated journalism moderates evaluations of algorithmically generated news. New Media Soc. 26, 5898–5922. doi: 10.1177/14614448221142534

[ref38] Jeng J. H. Kasangu G. Starke A. Trattner C. (2024). “Emotional reframing of economic news using a large language model,” in *Adjunct Proceedings of the 32nd ACM Conference on User Modeling, Adaptation and Personalization* (231–235).

[ref39] Jia C. (2020). Chinese automated journalism: a comparison between expectations and perceived quality. Int. J. Commun. 14, 22–22.

[ref40] Jia H. Appelman A. Wu M. Bien-Aime S. (2024). News bylines and perceived AI authorship: effects on source and message credibility. Comput. Hum. Behav. Artif. Hum. 2:100093. doi: 10.1016/j.chbah.2024.100093

[ref41] Jia C. Johnson T. J. (2021). Source credibility matters: does automated journalism inspire selective exposure? Int. J. Commun. 15, 22–22.

[ref42] Jia C. Liu R. (2021). Algorithmic or human source? Examining relative hostile media effect with a transformer-based framework. Media Commun. 9, 170–181. doi: 10.17645/mac.v9i4.4164

[ref43] Kim S. Kim B. (2020). A decision-making model for adopting al-generated news articles: preliminary results. Sustainability 12:7418. doi: 10.3390/su12187418

[ref44] Kohring M. Matthes J. (2007). Trust in news media: development and validation of a multidimensional scale. Commun. Res. 34, 231–252. doi: 10.1177/0093650206298071

[ref45] Krausová A. Moravec V. (2022). Disappearing authorship: ethical protection of AI-generated news from the perspective of copyright and other laws. J. Intell. Prop. Info. Tech. & Elec. Com. L. 13:132.

[ref46] Kreps S. McCain R. M. Brundage M. (2022). All the news that’s fit to fabricate: AI-generated text as a tool of media misinformation. J. Exp. Polit. Sci. 9, 104–117. doi: 10.1017/XPS.2020.37

[ref47] Lermann Henestrosa A. Greving H. Kimmerle J. (2023). Automated journalism: the effects of AI authorship and evaluative information on the perception of a science journalism article. Comput. Hum. Behav. 138:107445. doi: 10.1016/j.chb.2022.107445

[ref48] Lermann Henestrosa A. Kimmerle J. (2024). The effects of assumed AI vs. human authorship on the perception of a GPT-generated text. J. Media 5, 1085–1097. doi: 10.3390/journalmedia5030069

[ref49] Leuppert R. Weinmann C. Eiden J. (2025). AI-reporters as the future of journalism? Investigating recipients’ credibility evaluations of AI-authored news. Journalism. doi: 10.1177/14648849251382484

[ref50] Liu F. Chen M. Nah S. (2026). Who writes the news matters: the role of social trust in shaping credibility across AI, human and human–AI collaboration. Online Inf. Rev. 535, 1–19. doi: 10.1108/OIR-07-2025-0535

[ref51] Liu Y. Wang S. Yu G. (2023). The nudging effect of AIGC labeling on users’ perceptions of automated news: evidence from EEG. Front. Psychol. 14:1277829. doi: 10.3389/fpsyg.2023.1235043 38187414 PMC10766850

[ref52] Liu B. Wei L. (2019). Machine authorship in situ: effect of news organization and news genre on news credibility. Digital J. 7, 635–657. doi: 10.1080/21670811.2018.1510740

[ref53] Mayer R. C. Davis J. H. Schoorman F. D. (1995). An integrative model of organizational trust. Acad. Manag. Rev. 20, 709–734. doi: 10.2307/258792

[ref54] Metzger M. J. Flanagin A. J. (2013). Credibility and trust of information in online environments: the use of cognitive heuristics. J. Pragmat. 59, 210–220. doi: 10.1016/j.pragma.2013.07.012

[ref55] Moher D. Shamseer L. Clarke M. Ghersi D. Liberati A. Petticrew M. . (2015). Preferred reporting items for systematic review and meta-analysis protocols (PRISMA-P) 2015 statement. Syst. Rev. 4:1. doi: 10.1186/2046-4053-4-1, 25554246 PMC4320440

[ref56] Moravec V. Hynek N. Skare M. Gavurova B. Kubak M. (2024). Human or machine? The perception of artificial intelligence in journalism, its socio-economic conditions, and technological developments toward the digital future. Technol. Forecast. Soc. Change 200:123162. doi: 10.1016/j.techfore.2023.123162

[ref57] Page M. J. McKenzie J. E. Bossuyt P. M. Boutron I. Hoffmann T. C. Mulrow C. D. . (2021). The PRISMA 2020 statement: an updated guideline for reporting systematic reviews. BMJ 372, 1–15. 10.1136/bmj.n71 PMC8005924 33782057

[ref58] Popay J. Roberts H. Sowden A. Petticrew M. Arai L. Rodgers M. . (2006). Guidance on the conduct of narrative synthesis in systematic reviews. Prod. ESRC Methods Progr. Version 1:b92.

[ref59] Rae I. (2024). “The effects of perceived AI use on content perceptions,” in *Proceedings of the 2024 CHI Conference on Human Factors in Computing Systems* (1–14).

[ref60] Rethlefsen M. L. Kirtley S. Waffenschmidt S. Ayala A. P. Moher D. Page M. J. . (2021). PRISMA-S: an extension to the PRISMA statement for reporting literature searches in systematic reviews. Syst. Rev. 10:39. doi: 10.1186/s13643-020-01542-z, 33499930 PMC7839230

[ref61] Rossner A. Cassel M. Huschens M. (2024). “Do users really care? Evaluating the user perception of disclosing AI-generated content on credibility in (sports) journalism,” in *Proceedings of Mensch und Computer 2024* (413–418).

[ref62] Sadri S. R. Payne J. L. Brown K. A. Billings A. C. (2025). Sports news and the artificial-intelligence-generated article: examining identity and the influence of human versus artificial-intelligence authorship on perceptions of credibility and online share likelihood. Int. J. Sport Commun. 18, 160–174. doi: 10.1123/ijsc.2024-0208

[ref63] Schilke O. Reimann M. (2025). The transparency dilemma: how AI disclosure erodes trust. Organ. Behav. Hum. Decis. Process. 188:104405. doi: 10.1016/j.obhdp.2025.104405

[ref64] Schulz K. Rauenbusch J. Fillies J. Rutenburg L. Karvelas D. Rehm G. (2022) “User experience design for automatic credibility assessment of news content about COVID-19,” in *International Conference on Human-Computer Interaction* (142–165) Cham: Springer Nature Switzerland.

[ref65] Shi Y. Sun L. (2024). How generative AI is transforming journalism: development, application and ethics. J. Media 5, 582–594. doi: 10.3390/journalmedia5020039

[ref66] Shin S. Y. Lee J. (2022). The effect of deepfake video on news credibility and corrective influence of cost-based knowledge about deepfakes. Digit. Journal. 10, 412–432. doi: 10.1080/21670811.2022.2026797

[ref67] Spinde T. Wu F. Gaissmaier W. Demartini G. Echizen I. Giese H. (2025). Enhancing media literacy: the effectiveness of (human) annotations and bias visualizations on bias detection. Inf. Process. Manag. 62:104244. doi: 10.1016/j.ipm.2025.104244

[ref68] Sterne J. A. Hernán M. A. Reeves B. C. Savović J. Berkman N. D. Viswanathan M. . (2016). ROBINS-I: a tool for assessing risk of bias in non-randomised studies of interventions. BMJ 355:4919. doi: 10.1136/bmj.i4919 PMC5062054 27733354

[ref69] Sterne J. A. Savović J. Page M. J. Elbers R. G. Blencowe N. S. Boutron I. . (2019). RoB 2: a revised tool for assessing risk of bias in randomised trials. BMJ 366:l4898. doi: 10.1136/bmj.l4898 31462531

[ref70] Strikovic E. Cools H. (2025). Reality re-Imag (in) ed. mapping publics’ perceptions and evaluations of AI-generated images in news contexts. Digit. J. 22, 1–22. doi: 10.1080/21670811.2025.2573076

[ref71] Sun M. Hu W. Wu Y. (2024). Public perceptions and attitudes towards the application of artificial intelligence in journalism: from a China-based survey. Journal. Pract. 18, 548–570. doi: 10.1080/17512786.2022.2055621

[ref72] Sundar S. S. Kim J. (2019). “Machine heuristic: when we trust computers more than humans with our personal information,” in *Proceedings of the 2019 CHI Conference on human Factors in Computing Systems* (1–9).

[ref73] Tandoc E. C. Yao L. J. Wu S. (2020). Man vs. machine? The impact of algorithm authorship on news credibility. Digital J. 8, 548–562. doi: 10.1080/21670811.2020.1762102

[ref74] Tewari S. Zabounidis R. Kothari A. Bailey R. Alm C. O. (2021). Perceptions of human and machine-generated articles. Digital Threats Res. Prac. 2, 1–16. doi: 10.1145/3428158

[ref75] Toff B. Simon F. M. (2025). “Or they could just not use it?”: the dilemma of AI disclosure for audience trust in news. Int. J. Press/Politics 30, 881–903. doi: 10.1177/19401612241308697

[ref76] Vaccari C. Chadwick A. (2020). Deepfakes and disinformation: exploring the impact of synthetic political video on deception, uncertainty, and trust in news. Soc. Media Soc. 6:2056305120903408. doi: 10.1177/2056305120903408

[ref77] Velásquez-Salamanca D. Martín-Pascual M. Á. Andreu-Sánchez C. (2025). Interpretation of AI-generated vs. human-made images. Journal of. Imaging 11:227. doi: 10.3390/jimaging11070227, 40710614 PMC12295870

[ref78] Wischnewski M. Krämer N. (2024). Does polarizing news become less polarizing when written by an AI? Investigating the perceived credibility of news attributed to a machine in the light of the confirmation bias. J. Media Psychol. Theor. Methods Appl. 37, 388–399. doi: 10.1027/1864-1105/a000441

[ref79] Wittenberg C. Epstein Z. Péloquin-Skulski G. Berinsky A. J. Rand D. G. (2025). Labeling AI-generated media online. PNAS nexus 4:pgaf170. doi: 10.1093/pnasnexus/pgaf170, 40519990 PMC12166545

[ref80] Wölker A. Powell T. E. (2021). Algorithms in the newsroom? News readers’ perceived credibility and selection of automated journalism. Journalism 22, 86–103. doi: 10.1177/1464884918757072

[ref81] Wu Q. Li J. (2024). “Journalists' technological trust and willingness to use generative AI: a perspective based on risk perception theory,” in *2024 5th International Conference on Intelligent Computing and Human-Computer Interaction (ICHCI)* (526–530). IEEE.

[ref82] Yeste-Piquer E. Suau-Martínez J. Sintes-Olivella M. Xicoy-Comas E. (2025). What if i prefer robot journalists? Trust and objectivity in the AI news ecosystem. J. Media 6:51. doi: 10.3390/journalmedia6020051

[ref83] Zoizner A. Matthes J. Corbu N. de Vreese C. H. Esser F. Koc-Michalska K. . (2025). Can AI-attributed news challenge partisan news selection? Evidence from a conjoint experiment. Int. J. Press/Politics 19401612251342679, 1–26. doi: 10.1177/19401612251342679

