# Supplementary material for: When news is “written by artificial intelligence”: a systematic review of provenance and disclosure cues in journalism and their effects on credibility and trust
Source: Front Artif Intell. 2026 May 5;9:1815243. doi: 10.3389/frai.2026.1815243 (PMC13183635; doi:10.3389/frai.2026.1815243)
Supplement: Supplementary file 4 [file Table_4.DOCX]

## Appendix D: Characteristics of included full-text studies (N = 47)

Table D1. Characteristics of included full-text studies (N = 47): broad design classification, sample descriptor, AI exposure type, outcome domain/target, and extracted direction-of-effect coding.

| **ID** | **Study** | **Design & sample** | **Exposure(s)** | **Outcomes** | **Direction** |
| --- | --- | --- | --- | --- | --- |
| 1 | Wölker & Powell (2021) Algorithms in the newsroom? News readers’ perceived credibility and selection of automated journalism | Online experiment (between-subjects source x within-subjects topic); 300; Europe (European news readers; multi-country; recruited via social media) (Recruitment: Snowball sampling via Facebook/Twitter/LinkedIn; filtered to European nationality) | Provenance/authorship | Credibility + Trust; Message/article; Source/journalist; Outlet | Cred: Positive; Trust: NR |
| 3 | Blom et al. (2025) “I Think It’s Exciting and Frightening at the Same Time”: Audience Sentiments Toward the Use and Labeling of Generative AI in Journalism | Qualitative semi-structured interviews (two-wave design); 31; Denmark (Recruitment: Purposive sampling for diversity (gender, age, region, education, AI experience)) | Combined (provenance + disclosure) | Unclear; Message/article; Source/journalist | Cred: NR; Trust: NR; Disc: NR |
| 6 | Haim & Maurus (2023) Stereotypes and sexism? Effects of gender, topic, and user comments on journalists’ credibility | Online between-subjects experiment (3 author x 2 topic x 2 sexist comments; computer author only with non-sexist comments → 10 groups); 417; Germany/Austria/Switzerland (German-speaking participants) (Recruitment: SoSci Panel (online-access convenience panel); invitations to 2000 panelists; final N=417 after exclusions) | Provenance/authorship | Credibility; Message/article; Source/journalist | Cred: Negative; Trust: NR |
| 8 | Liu et al. (2026) Who writes the news matters: the role of social trust in shaping credibility across AI, human and human-AI collaboration | Online survey with within-respondent ratings of credibility across authorship types; CB-SEM moderation model; 1252; United States (Dynata panel; targeted to mirror US adult demographics) (Recruitment: Dynata panels; quota sampling for representativeness; fielded Apr 27–May 6, 2022 (pre-ChatGPT release)) | Provenance/authorship | Credibility + Trust; Message/article | Cred: Mixed/conditional; Trust: Mixed/conditional |
| 9 | Jia & Liu (2021) Algorithmic or human source? Examining relative hostile media effect with a transformer‐based framework | Online between-subjects experiment (author/source attribution) + within-subjects headlines (pro/anti/neutral); 511; United States (Recruitment: CloudResearch/TurkPrime; US adults 18+; HIT approval >95%; paid $0.75; N=511 after exclusions) | Provenance/authorship | Credibility; Source/journalist | Cred: Mixed/conditional; Trust: NR |
| 11 | Jia (2020) Chinese Automated Journalism: A Comparison Between Expectations and Perceived Quality | Two online experiments / survey-experiments comparing automated vs human-written news; Study 1 mixed (source between-subject; topic within-subject); Study 2 within-subject (expectations + perceived quality); Study 1: N=125 (online participants, Feb 2019); Study 2: N=308 (online participants, Apr 2018); China (Recruitment: Convenience online recruitment via Chinese social media) | Provenance/authorship | Credibility; Unclear | Cred: No effect; Trust: NR |
| 15 | Strikovic & Cools (2025) Reality Re-Imag(in)ed. Mapping Publics’ Perceptions and Evaluations of AI-Generated Images in News Contexts | Qualitative focus groups exploring perceptions and evaluations of AI-generated images used in news contexts; 4 focus groups; N=25 (mixed-gender; varied ages; recruited via private online company); Netherlands (Recruitment: Recruitment via an online panel/research company in the Netherlands) | Provenance/authorship | Credibility + Trust; Message/article; Outlet | Cred: NR; Trust: NR |
| 17 | Forstner et al. (2025) Evaluating Image Trust Labels in a News Recommender System | Between-subject online experiment in a simulated news recommender system with 4 label conditions (3 provenance/verification labels vs baseline no-label); N=202 (online participants); United Kingdom and United States (Recruitment: Online participants (country: UK/US; panel not specified in extracted text)) | Combined (provenance + disclosure) | Trust; Message/article; Source/journalist | Cred: NR; Trust: No effect; Disc: NR |
| 20 | Lermann Henestrosa et al. (2023) Automated journalism: The effects of AI authorship and evaluative information on the perception of a science journalism article | Three preregistered online experiments: S1 2(authorship: AI vs human)×2(info: neutral vs evaluative-positive) between + topic within; S2 2×3(authorship×info: neutral/positive/negative) between; S3 2×2 between (authorship×info).; 715; German-speaking participants (online; recruited via Prolific) (Recruitment: Prolific (German speakers; attention checks and exclusions applied).) | Provenance/authorship | Credibility + Trust; Message/article; Source/journalist | Cred: No effect; Trust: No effect |
| 22 | Gavurova et al. (2024) An information-analytical system for assessing the level of automated news content according to the population structure – A platform for media literacy system development | Cross-sectional survey + two-stage information–analytical system (information model + multicriteria fuzzy model) to aggregate attitudes toward artificially created/automated news content into linguistic levels (d1–d5).; 1041; Czech Republic (citizens surveyed; April 4–11, 2023) (Recruitment: Survey of Czech citizens (sampling/recruitment not fully specified in the PDF text extraction; reports N=1041 and dates Apr 4–11, 2023).) | Unclear | Credibility + Trust; Message/article | Cred: NR; Trust: NR |
| 23 | Moravec et al. (2024) Human or machine? The perception of artificial intelligence in journalism, its socio-economic conditions, and technological developments toward the digital future | Representative online survey with embedded tasks: (RQ1) identify AI- vs human-written news text pairs; (RQ2) rate authenticity of AI-generated vs human-read traffic-report audio; (RQ3) compare views on journalism’s future among ChatGPT users vs non-users.; 1041; Czech Republic (statistically representative CAWI sample; Apr 4–11, 2022; data gathered with IPSOS) (Recruitment: Computer-assisted web interviews (CAWI) via Charles University + IPSOS; stratified by age, gender, education, region, and settlement size; ~10 min structured questionnaire.) | Provenance/authorship | Credibility + Trust; Source/journalist | Cred: NR; Trust: NR |
| 27 | Wischnewski & Krämer (2025) Does Polarizing News Become Less Polarizing When Written by an AI? Investigating the Perceived Credibility of News Attributed to a Machine in the Light of the Confirmation Bias | Preregistered 2×2 between-subjects online experiment: author attribution (machine vs human) × attitude confirmation (confirming vs disconfirming article relative to participant attitude).; N=508 (female=258; male=243; nonbinary=4; prefer not=3); Online sample recruited via Prolific (demographics reported; mean age ≈29.7; relatively educated). (Recruitment: Prolific online panel; ethics approval noted; preregistration on OSF.) | Provenance/authorship | Credibility + Trust; Message/article; Source/journalist | Cred: No effect; Trust: No effect |
| 28 | Sadri et al. (2025) Sports News and the Artificial-Intelligence-Generated Article: Examining Identity and the Influence of Human Versus Artificial-Intelligence Authorship on Perceptions of Credibility and Online Share Likelihood | 3×2 factorial design: authorship (Associated Press human vs ChatGPT vs Google Gemini) × byline cue (human byline vs AI byline). Between-subjects exposure to one article condition.; N=355 valid surveys; United States (nationwide adult sample recruited via Prolific). (Recruitment: Prolific online panel; survey built in Qualtrics; compensated (~$3).) | Combined (provenance + disclosure) | Credibility; Message/article; Source/journalist | Cred: NR; Trust: NR; Disc: NR |
| 32 | Tewari et al. (2021) Perceptions of Human and Machine-Generated Articles | Lab experiment with within-subject exposure to human-written vs GPT-2-generated news-like article excerpts across political leaning (conservative vs liberal) and three topics; collects self-reports plus biometric measures (facial expressions, galvanic skin response).; n=30 participants (each read 12 article excerpts; total ratings N=359 datapoints); United States (laboratory study; participants recruited via flyers/emails). (Recruitment: Flyers and email recruitment; IRB approval; $10 compensation.) | Provenance/authorship | Credibility; Message/article; Source/journalist | Cred: NR; Trust: NR |
| 34 | Rae (2024) The Effects of Perceived AI Use On Content Perceptions | 3 (assigned creator: human vs human+AI assistance vs AI; within-subjects) × 4 (context: news/travel/health/jokes; between-subjects) mixed-design experiment; all stimuli human-written but randomly labeled as human/AI-assisted/AI-generated.; Main experiment: n=1,641 valid responses (across news/travel/health/jokes contexts); United States (Cint panel; representative gender/age; online survey). (Recruitment: Anonymous Cint survey panel; administered via Qualtrics; compensated; exclusions for low-quality responses.) | Provenance/authorship | Credibility; Message/article; Source/journalist | Cred: NR; Trust: NR |
| 35 | Rossner et al. (2024) Do Users Really Care? Evaluating the User Perception of Disclosing AI-Generated Content on Credibility in (Sports) Journalism | Online survey experiment: between-subject disclosure condition (no disclosure vs correct disclosure vs manipulated disclosure); within-subject evaluation of 2 match reports (human-written then AI-generated).; 154; Germany (online sample; German-language study) (Recruitment: Not clearly reported (online convenience sample).) | Combined (provenance + disclosure) | Credibility + Trust; Message/article; Source/journalist | Cred: No effect; Trust: No effect; Disc cred: No effect |
| 39 | Heiselberg et al. (2022) Automated News Reading in the Neural Age: Audience Reception and Perceived Credibility of a News Broadcast Read By a Neural Voice | Qualitative reception analysis: participants listened to a full radio news broadcast read by a neural voice; interviews conducted first unprimed (no mention of automation) then primed (told the voice was artificial).; 12; Denmark (Danish radio listeners). (Recruitment: Recruitment via Wilke A/S online panel; screened for Danish radio listeners.) | Provenance/authorship | Credibility; Message/article; Outlet | Cred: NR; Trust: NR |
| 41 | Bykov & Kurushkin (2025) Communication Strategy of AI-application in News Reporting: Human Values or Technological Effectiveness? | Online survey: participants evaluated/identified authorship of news materials generated by ChatGPT vs written by journalists, and rated attitudes toward AI application in news reporting.; 108; Not specified (pilot survey; likely Russian-speaking respondents). (Recruitment: Not reported (pilot study sample).) | Provenance/authorship | Trust; Unclear | Cred: NR; Trust: Negative |
| 42 | Haim et al. (2025) Contextual Changes, Credible Conclusions? A Direct and Conceptual Replication of Shen et al.'s (2019) Study on Online Image Credibility | Two online experimental replications of Shen et al. (2019): direct replication using original manually fabricated images (US sample) and conceptual replication using AI-generated fake images and German-context adaptations (German sample).; Study 1 n=634; Study 2 n=513; Study 1: USA (MTurk via CloudResearch); Study 2: Germany (SoSci Panel). (Recruitment: Study 1: MTurk participants recruited via CloudResearch; Study 2: German-language SoSci Panel (academic convenience panel).) | Provenance/authorship | Credibility; Source/journalist | Cred: NR; Trust: NR |
| 44 | Graefe et al. (2018) Readers’ Perception of Computer-generated News: Credibility, Expertise, and Readability | Online experiment manipulating actual source (computer-generated vs journalist-written) and declared source/byline (computer vs journalist) across two news topics.; 986; Germany (online panel; SoSci Panel). (Recruitment: SoSci Panel (academic convenience online panel).) | Provenance/authorship | Credibility; Message/article; Source/journalist | Cred: Negative; Trust: NR |
| 45 | Jang et al. (2024) Knowledge of automated journalism moderates evaluations of algorithmically generated news | Two online experiments. Exp1: 2 (declared story source: journalist vs robot reporter) × 2 (algorithm description: human-like vs machine-like) between-subjects; 3 within-subject story topics (baseball/football/basketball). Exp2: 2 (declared story source: journalist vs robot reporter) × 2 (AJ knowledge explanation vs control) between-subjects; 3 within-subject story topics.; Exp1 n=101; Exp2 n=220 (total n=321; after exclusions where applicable); United States (online sample; Prolific) (Recruitment: Online recruitment via Prolific (U.S. participants).) | Provenance/authorship | Credibility; Source/journalist | Cred: NR; Trust: NR |
| 46 | Krausová & Moravec (2022) Disappearing Authorship: Ethical Protection of AI-Generated News from the Perspective of Copyright and Other Laws | Primarily normative legal/ethical analysis, complemented by a pilot qualitative study (semi-structured interviews) exploring newsroom professionals’ perceptions of AI-generated news, authorship, responsibility, and disclosure practices (incl. comparison across two Czech news organizations).; Pilot qualitative study: n=20 semi-structured interviews (10 editors at E15; 10 journalists at ČTK); Czech Republic (newsroom professionals: editors at E15; journalists at Czech News Agency/ČTK) (Recruitment: Purposive recruitment of newsroom professionals from E15 (editors) and ČTK (journalists); interview dates reported (E15: Sep 2020; ČTK: Feb 2021).) | Provenance/authorship | Trust; Message/article | Cred: NR; Trust: NR |
| 49 | Kreps et al. (2022) All the News That’s Fit to Fabricate: AI-Generated Text as a Tool of Media Misinformation | Three online experiments evaluating human-written baseline vs GPT-2–generated news (varying model sizes). Exp1 compared perceived credibility and ability to detect AI text across model sizes vs a human baseline. Exp2 used a 3×3 design manipulating story ideology (left/center/right) and story source (human original vs AI-generated vs AI-generated + disclaimer). Exp3 tested credibility evaluations for AI-generated stories from three model sizes (no human baseline in that experiment).; Three experiments; Exp1: n≈1,512 (3 AI model-size groups); Exp2: >1,500; Exp3: n=600 (200 per AI model-size condition); United States (online participants via Amazon Mechanical Turk). (Recruitment: Amazon Mechanical Turk (U.S. adult participants).) | Provenance/authorship | Credibility; Message/article | Cred: NR; Trust: NR |
| 51 | Schulz et al. (2022) User Experience Design for Automatic Credibility Assessment of News | Between-subjects experiment with 3 conditions: (1) control/no automatic credibility assessment (ACA), (2) ACA with bar-chart explanation, (3) ACA with textual explanation. Participants judged credibility of 12 COVID-19 news items (mixture of true and false) and reported confidence, agreement, and perceptions of the system.; N=141 participants (experiment); Not explicitly stated in provided excerpt (online participants; study conducted in English; recruitment platform not specified in extracted snippets). (Recruitment: Online user study (recruitment platform not specified in extracted snippets).) | Provenance/authorship | Credibility + Trust; Message/article; Source/journalist | Cred: Mixed/conditional; Trust: Mixed/conditional |
| 53 | Zoizner et al. (2025) Can AI-Attributed News Challenge Partisan News Selection? Evidence from a Conjoint Experiment | Preregistered conjoint experiment simulating a social-media feed with high-choice environment. Participants completed five choice tasks comparing pairs of news posts/headlines. Manipulated attributes include: source label (Fox News, MSNBC, CBS News, fictitious “AI News”), authorship attribution (human author vs AI vs no author disclosed), political slant (pro-left/pro-right/neutral), issue type (polarized vs non-polarized topics), and social engagement levels (likes/comments/shares).; N=2,011; United States (Prolific sample; partisan identifiers; pure independents excluded). (Recruitment: Prolific Academic (U.S. respondents), data collected Sept 24–26, 2024 (per notes).) | Combined (provenance + disclosure) | Credibility + Trust; Message/article; Outlet | Cred: NR; Trust: Negative; Disc: N/A |
| 54 | Spinde et al. (2025) Enhancing media literacy: The effectiveness of (Human) annotations and bias visualizations on bias detection | Two online experiments with a teaching phase (annotated/visualized text) and a testing phase (unlabeled/new-topic text). Between-subjects assignment to label/visualization conditions to test learning and generalization after intervention removal.; Study 1: N=470 analyzed (512 recruited). Study 2: N=846 analyzed (1,121 recruited); Study 1: Prolific (US + UK; representative sample by age/sex/ethnicity). Study 2: Prolific (US residents; balanced gender; high approval). (Recruitment: Prolific (Study 1 representative quotas for sex/age/ethnicity; US/UK). Prolific (Study 2: US residency + English fluency; paid).) | Combined (provenance + disclosure) | Credibility; Unclear | Cred: Mixed/conditional; Trust: NR; Disc cred: Mixed/conditional |
| 55 | Danry et al. (2025) Deceptive Explanations by Large Language Models Lead People to Change their Beliefs About Misinformation More Often than Honest Explanations | Preregistered online experiment: participants rated the truth of 20 statements, received AI feedback (classification-only vs classification+explanation), and could revise beliefs. Mixed design with between-subjects statement domain (news headlines vs trivia) and feedback type, and within-study variation in honest vs deceptive explanations and logical validity.; N=589 analyzed (596 recruited); 11,780 headline observations (23,980 total observations incl. trivia); Prolific (US citizens). (Recruitment: Prolific; US citizens; compensated; demographic exclusions reported.) | Provenance/authorship | Credibility; Message/article; Source/journalist | Cred: Negative; Trust: NR |
| 56 | Govers et al. (2025) Feeds of Distrust: Investigating How AI-Powered News Chatbots Shape User Trust and Perceptions | Online experiment simulating chatbot-augmented news across four topics. 2 (news stance: pro/anti) × 3 (chatbot stance: pro/anti/control without chatbot) design, repeated within-subjects across topics with counterbalancing to ensure each participant experienced congruent, incongruent, and control conditions.; N=100; Prolific (US-based participants). (Recruitment: Prolific; US location; balanced gender and political orientation via filters; first-language English speakers.) | Provenance/authorship | Credibility + Trust; Message/article; Source/journalist | Cred: Negative; Trust: NR |
| 57 | La-Rosa & SANDOVAL-MARTIN (2024) Artificial intelligence versus journalists: The quality of automated news and bias by authorship using a Turing test | Online Turing-test experiment with 2×2 between-subjects manipulation: Real authorship (human journalist vs automated NLG) × Declared authorship label (human journalist vs AI).; N=128 analyzed (balanced 32 per condition; N=222 initial); Spain (mostly Spanish communication/journalism students; mean age ≈21.7). (Recruitment: Survey links distributed via Universidad Carlos III de Madrid journalism program networks (students/staff sharing links); May 2020.) | Provenance/authorship | Credibility; Source/journalist | Cred: No effect; Trust: NR |
| 58 | Chen et al. (2025) Generative AI in the News: The Impact of Framing on Public Attitude and Engagement | Computational content analysis: topic modeling (LDA) and framing analysis of news headlines vs Facebook post descriptions about generative AI; sentiment/emotion analysis of user comments; regression models predicting engagement metrics by outlet ideology and framing.; 550 Facebook posts (headlines + descriptions) and 34,098 user comments (collected 11/30/2022–3/08/2024); United States (Facebook pages of major liberal and conservative news outlets; audience reactions/comments). (Recruitment: N/A (archival social media data via CrowdTangle and Facebook scraping).) | Provenance/authorship | Trust; Message/article; Source/journalist | Cred: No effect; Trust: No effect |
| 59 | García-Marín et al. (2025) “Everything is believable”. Credibility of disinformation produced by using AI and the perception of Spanish communication students | Controlled classroom survey (within-subjects): participants rated 6 short news items on truthfulness, accuracy, and clarity (1–5 Likert). Items included AI-generated fake news (ChatGPT 3.5) with and without minimal human post-editing, plus true agency news; no disclosure that items mixed true and false content.; N=245 students; Spain (communication students at Rey Juan Carlos University; classroom-controlled administration). (Recruitment: Convenience sample of communication students; administered on multiple days (9 Feb–3 Mar 2024) in classrooms; monitored to prevent web searching.) | Provenance/authorship | Credibility; Unclear | Cred: Positive; Trust: NR |
| 60 | Gherheş et al. (2025) AI vs. Human-Authored Headlines: Evaluating the Effectiveness, Trust, and Linguistic Features of ChatGPT-Generated Clickbait and Informative Headlines in Digital News | Online questionnaire: participants evaluated headline triplets for 100 Romanian news articles (original human headline + ChatGPT-3.5 clickbait version + ChatGPT-3.5 informative version). Each participant rated one randomly assigned set of 10 triplets; authorship was blinded (no indication that 2/3 were AI-generated). Includes linguistic analysis of preferred headline types.; N=624 students (18–24); Romania (university students; Timisoara). (Recruitment: University students; invitations distributed by research team; online platform; data collected 25 Sep–20 Oct 2024; voluntary/no incentives.) | Provenance/authorship | Credibility + Trust; Message/article; Source/journalist | Cred: Negative; Trust: Negative |
| 61 | Velásquez-Salamanca et al. (2025) Interpretation of AI-Generated vs. Human-Made Images | Online survey experiment: within-subjects exposure to 32 images (4 categories) in randomized order. Participants rated (1) believed source (AI vs human), (2) realism, and (3) credibility. Between-group comparison by visual professionalization (visual professionals vs non-professionals).; N=161 participants; 32 images (24 AI-generated, 8 human-made); 5152 evaluations per question; Online sample (country not specified); includes 71 visual professionals and 90 non-professionals. (Recruitment: Online survey distributed 16 Apr–18 Jun 2024; recruitment platform not specified; informed consent obtained.) | Provenance/authorship | Credibility; Message/article; Source/journalist | Cred: Positive; Trust: NR |
| 62 | Wu & Li (2024) Journalists' Technological Trust and Willingness to Use Generative AI: A Perspective Based on Risk Perception Theory | Cross-sectional survey; confirmatory factor analysis (CFA) + structural equation modelling (SEM) testing perceived risk dimensions → technological trust → intention to use GenAI.; N=357 valid responses (400 distributed); Journalists/editors (sampling country not explicitly stated; authors affiliated with China). (Recruitment: Online questionnaire distributed to journalism/media professionals via an online platform.) | Provenance/authorship | Trust; Message/article | Cred: NR; Trust: NR |
| 64 | Cools & Diakopoulos (2024) Uses of Generative AI in the Newsroom: Mapping Journalists’ Perceptions of Perils and Possibilities | Qualitative study: semi-structured interviews with journalists; thematic content analysis (open + axial coding) in NVivo; maps 16 use cases across news reporting process.; 15; The Netherlands and Denmark (journalists from NOS, NRC, and Berlingske Media; early adopters of GenAI tools). (Recruitment: Convenience sampling via prior access to 3 legacy outlets; key contacts invited by email and asked to suggest additional respondents (snowball).) | Provenance/authorship | Credibility; Source/journalist; Outlet | Cred: NR; Trust: NR |
| 65 | Jia & Johnson (2021) Source Credibility Matters: Does Automated Journalism Inspire Selective Exposure? | Mixed-design online experiment: 2(author attribution: human vs algorithm; between) × 3(article attitude: attitude-consistent vs attitude-challenging vs neutral; within) × 2(topic: gun control vs abortion; within). Measures include selection (selective exposure/avoidance), message credibility, and source credibility; mediation tested.; 351; United States (MTurk sample; online experiment conducted in 2019). (Recruitment: Amazon Mechanical Turk (US participants); paid $0.80; attention checks; exclusions for <18, repeated IPs, incomplete, failed checks.) | Provenance/authorship | Credibility; Message/article; Source/journalist | Cred: Mixed/conditional; Trust: NR |
| 69 | Dobber et al. (2025) A Beacon of Trustworthiness in a Sea of Disinformation: Does News Coverage About the Dangers of Generative AI Cause People to Flock to Journalism? | Preregistered between-subjects online experiment with 5 GenAI-coverage framing treatments (neutral, alarmist, relativizing, gain, loss) + control article; random assignment.; 658; Netherlands (Dutch adults; online experiment; recruited via Dynata panel; June 6–9, 2023). (Recruitment: Dynata (survey company/panel; Netherlands); incentive €2.50; speeders/flatliners excluded.) | Provenance/authorship | Credibility + Trust; Message/article | Cred: Positive; Trust: Negative |
| 70 | Yeste-Piquer et al. (2025) What If I Prefer Robot Journalists? Trust and Objectivity in the AI News Ecosystem | Qualitative focus-group study (5 groups; ~2 hours each) exploring (1) AI knowledge/use, (2) AI in journalism (trust/objectivity), and (3) detection of AI-manipulated video; sessions recorded and transcribed for content analysis.; 39; Spain (Barcelona metropolitan area); 5 focus groups (June 25–July 4, 2024) with diverse ages (18–50) and AI-use levels; 50/50 gender split. (Recruitment: Purposive selection from GESOP panel (Barcelona market research firm) based on age, gender, news consumption, and AI tool use.) | Provenance/authorship | Credibility + Trust; Message/article | Cred: No effect; Trust: No effect |
| 73 | DeVerna et al. (2024) Fact-checking information from large language models can decrease headline discernment | Preregistered randomized online experiment; between-subjects 4 (fact-check condition: control vs LLM-optional vs LLM-forced vs human fact-check) × 2 (task: belief vs sharing). Repeated measures across 40 headlines.; N=2,159; United States (quota-matched online sample). (Recruitment: Qualtrics online panel; quota-based sampling to approximate U.S. Census distributions (state, age, gender, race/ethnicity).) | Combined (provenance + disclosure) | Credibility; Message/article; Source/journalist | Cred: Positive; Trust: NR; Disc cred: Positive |
| 74 | Sun et al. (2024) Public Perceptions and Attitudes Towards the Application of Artificial Intelligence in Journalism: From a China-based Survey | Cross-sectional online survey (public perceptions of AI in journalism).; N=1,558 (valid responses after excluding incomplete/invalid); China (mainland). (Recruitment: Sojump.com online survey panel; random sampling from enrollees; respondents with basic understanding of AI (self-reported).) | Combined (provenance + disclosure) | Credibility; Message/article | Cred: Positive; Trust: NR; Disc: N/A |
| 75 | Cloudy et al. (2023) The Str(AI)ght Scoop: Artificial Intelligence Cues Reduce Perceptions of Hostile Media Bias | Online between-subjects experiment manipulating journalist source identity (human journalist vs AI ‘NewsBot’) in a social media news post.; N=255; Not reported (online sample recruited via Prolific; screened for partisan identity). (Recruitment: Prolific panels; issue-specific sampling criterion used to recruit Democrats and Republicans; compensation via Prolific.) | Provenance/authorship | Credibility; Message/article; Source/journalist | Cred: Mixed/conditional; Trust: NR |
| 77 | Jeng et al. (2024) Emotional Reframing of Economic News using a Large Language Model | Online between-subjects experiment (4 framing conditions) using LLM-based emotional reframing of economic news summaries.; N=200; Not reported (online experiment; location not stated in accessible excerpt). (Recruitment: Not reported (full text needed for recruitment platform and sampling frame).) | Provenance/authorship | Credibility + Trust; Source/journalist | Cred: NR; Trust: NR |
| 80 | Gong (2023) AI voices reduce cognitive activity? A psychophysiological study of the media effect of AI and human newscasts in Chinese journalism | Psychophysiological experiment: pre-experiment within-subject (AI vs human voice) + formal 2 (agent: AI vs human, between) × 2 (valence: emotional vs neutral, within) mixed factorial with EEG.; N=30 (formal experiment); pre-experiment within-subject subset n=10; China (lab-based study; author affiliation Fudan University, Shanghai). (Recruitment: Lab recruitment (details not specified; participants compensated; screened for hearing/vision, neurological/psychiatric conditions).) | Provenance/authorship | Credibility + Trust; Message/article; Source/journalist | Cred: Mixed/conditional; Trust: Mixed/conditional |
| 81 | Kim & Kim (2020) A Decision-Making Model for Adopting Al-Generated News Articles: Preliminary Results | Decision-making model development using AHP; survey/interview-based weighting of factors affecting adoption of AI-generated news articles.; N=30 (15 journalists + 15 AI engineers); Korea (participants from six major Korean media outlets). (Recruitment: Participants from six major Korean media outlets; two groups: journalists and AI engineers; surveys conducted via one-on-one interviews with questionnaires.) | Provenance/authorship | Trust; Message/article | Cred: NR; Trust: NR |
| 84 | Lermann Henestrosa & Kimmerle (2024) The Effects of Assumed AI vs. Human Authorship on the Perception of a GPT-Generated Text | Preregistered online between-subjects experiment (1-factor: labeled authorship AI vs human) using identical GPT-3–generated science journalism article; equivalence testing planned.; 734; German-speaking adult sample (recruited via Prolific; German language requirement) (Recruitment: Prolific (German-speaking; age ≥18); compensated ~£1.25; preregistered exclusions applied.) | Combined (provenance + disclosure) | Credibility; Message/article; Source/journalist | Cred: No effect; Trust: NR; Disc cred: No effect |
| 85 | Liu & Wei (2019) Machine Authorship In Situ: Effect of news organization and news genre on news credibility | 2 (purported writer: human vs machine) × 2 (news organization: New York Times vs Fox News) × 2 (news type: spot vs interpretive) between-subjects online experiment.; 573 recruited (US MTurk); final N=355 after topic/source/author ID checks + attention check exclusions; United States (Amazon Mechanical Turk participants). (Recruitment: Amazon Mechanical Turk (US); participants compensated $0.25.) | Provenance/authorship | Credibility + Trust; Outlet | Cred: Mixed/conditional; Trust: Mixed/conditional |
| 86 | Fernández-Barrero & Serrano-Martín (2025) Are the Media Transparent in Their Use of AI? Self-Regulation and Ethical Challenges in Newsrooms in Spain | Mixed methods: quantitative survey + qualitative in-depth interviews on newsroom AI use, ethics, and transparency practices.; Survey: N=50 journalists. Interviews: n=8 in-depth interviews (mixed methods design); Spain (journalists working in newsrooms across various Spanish provinces). (Recruitment: Survey sample selected via random sampling (as reported) across Spanish provinces; interviews with journalists representing diverse perspectives.) | Disclosure cues | Credibility + Trust; Message/article | Cred: Mixed/conditional; Trust: Mixed/conditional; Disc cred: Mixed/conditional |

Note. ID corresponds to the included-study record_id used in Appendix A (sheet “included for analysis fulltext”) and Appendix B (sheet “included_fulltext_unique”). The PRISMA master list uses prisma_record_id. Direction coding is based on extractable comparisons reported in each paper (Cred = credibility; Trust = trust; Disc = disclosure effect where applicable; NR = not reported/extractable; N/A = not applicable).

## Appendix E: Quality appraisal notes (MMAT-informed)

Table E1. Study-level quality appraisal notes (MMAT-informed): descriptive strengths/limitations used to contextualise confidence in findings (not numerical scores).

| **ID** | **Study** | **Design** | **Appraisal notes** |
| --- | --- | --- | --- |
| 1 | Wölker & Powell (2021) Algorithms in the newsroom? News readers’ perceived credibility and selection of automated journalism | Experiment | Internal validity good; random assignment implied; source credibility not measured for control condition. |
| 3 | Blom et al. (2025) “I Think It’s Exciting and Frightening at the Same Time”: Audience Sentiments Toward the Use and Labeling of Generative AI in Journalism | Qualitative | Qualitative; strong for interpretive insight into labeling but not causal effects. |
| 6 | Haim & Maurus (2023) Stereotypes and sexism? Effects of gender, topic, and user comments on journalists’ credibility | Survey | Preregistered; OSF materials/data provided; computer-author not crossed with sexist comments (design constraint). |
| 8 | Liu et al. (2026) Who writes the news matters: the role of social trust in shaping credibility across AI, human and human-AI collaboration | Unclear/other | Large quota-based panel; observational ratings (not randomized exposure to actual articles). |
| 9 | Jia & Liu (2021) Algorithmic or human source? Examining relative hostile media effect with a transformer‐based framework | Experiment | Random assignment; attention checks; strong reporting; credibility measured on 7-point scale. |
| 11 | Jia (2020) Chinese Automated Journalism: A Comparison Between Expectations and Perceived Quality | Unclear/other | Strong for separating expectations vs perceptions and comparing within vs between exposure; however, no explicit disclosure manipulation, and stimuli were limited to selected genres. |
| 15 | Strikovic & Cools (2025) Reality Re-Imag(in)ed. Mapping Publics’ Perceptions and Evaluations of AI-Generated Images in News Contexts | Unclear/other | Rich qualitative evidence for mechanisms and expectations; does not provide causal estimates of disclosure/provenance effects. |
| 17 | Forstner et al. (2025) Evaluating Image Trust Labels in a News Recommender System | Experiment | Well-aligned to disclosure-cue research; clear manipulation and multiple outcomes; trust measured at both image and article levels; comprehension issues highlight construct validity considerations. |
| 20 | Lermann Henestrosa et al. (2023) Automated journalism: The effects of AI authorship and evaluative information on the perception of a science journalism article | Unclear/other | Strengths: preregistered multi-study design; random assignment; replication across 3 experiments. Limitation: authorship was manipulated (texts not necessarily AI-generated); generalizability limited to German-speaking online sample and science journalism stimulus. |
| 22 | Gavurova et al. (2024) An information-analytical system for assessing the level of automated news content according to the population structure – A platform for media literacy system development | Unclear/other | Large dataset and explicit modeling framework; however, not a direct audience-credibility experiment and some sampling/measurement details are limited in the extracted text. Best suited for media-literacy/attitude synthesis rather than causal effects on credibility. |
| 23 | Moravec et al. (2024) Human or machine? The perception of artificial intelligence in journalism, its socio-economic conditions, and technological developments toward the digital future | Unclear/other | Large, stratified national sample; uses realistic local news stimuli and both text + audio. Limitations: identification task is not naturalistic ‘byline disclosure’ (participants explicitly primed to detect AI); outcomes focus on recognition/attitudes rather than message credibility or trust scales. |
| 27 | Wischnewski & Krämer (2025) Does Polarizing News Become Less Polarizing When Written by an AI? Investigating the Perceived Credibility of News Attributed to a Machine in the Light of the Confirmation Bias | Experiment | Strengths: preregistered design; decomposes source credibility (trustworthiness vs expertise) and measures affect. Limitations: single topic/issue; machine attribution (not necessarily actual AI-written text) which may understate real AIGC differences. |
| 28 | Sadri et al. (2025) Sports News and the Artificial-Intelligence-Generated Article: Examining Identity and the Influence of Human Versus Artificial-Intelligence Authorship on Perceptions of Credibility and Online Share Likelihood | Survey | Strengths: compares two contemporary LLMs (ChatGPT vs Gemini) plus human source; manipulates byline separately from authorship; includes identity/attitudinal moderators. Limitations: single sports topic; self-report outcomes; Prolific panel may not generalize to all news audiences. |
| 32 | Tewari et al. (2021) Perceptions of Human and Machine-Generated Articles | Unclear/other | Strengths: multi-method (self-report + physiological measures) and uses real GPT-2 generation with political topics. Limitations: small lab sample (n=30); GPT-2 2019 generation quality may differ from current LLMs; no real-world byline/disclosure context. |
| 34 | Rae (2024) The Effects of Perceived AI Use On Content Perceptions | Experiment | Strengths: large sample; controls content by keeping text constant while varying labels; combines quantitative and qualitative evidence. Limitations: multi-context (not exclusively news); uses labels rather than actual AI-generation differences in text. |
| 35 | Rossner et al. (2024) Do Users Really Care? Evaluating the User Perception of Disclosing AI-Generated Content on Credibility in (Sports) Journalism | Survey | Short conference paper; recruitment/source population unclear; fixed order (human then AI) may introduce order effects; single sports topic limits generalizability. |
| 39 | Heiselberg et al. (2022) Automated News Reading in the Neural Age: Audience Reception and Perceived Credibility of a News Broadcast Read By a Neural Voice | Qualitative | Rich qualitative insights but small sample (n=12) and Denmark-specific radio context; disclosure manipulation occurred within interview setting rather than in naturalistic exposure. |
| 41 | Bykov & Kurushkin (2025) Communication Strategy of AI-application in News Reporting: Human Values or Technological Effectiveness? | Survey | Brief conference paper; limited methodological detail (recruitment, scales) and unclear stimulus exposure procedure. |
| 42 | Haim et al. (2025) Contextual Changes, Credible Conclusions? A Direct and Conceptual Replication of Shen et al.'s (2019) Study on Online Image Credibility | Unclear/other | Strong open-science practices (materials/data); complex multi-condition design; AI component is limited to stimulus generation (not an AI-label/disclosure test). |
| 44 | Graefe et al. (2018) Readers’ Perception of Computer-generated News: Credibility, Expertise, and Readability | Experiment | Early foundational study; uses algorithmic news generation in constrained domains (sports/finance) and non-probability online panel; disclosure is operationalized via byline rather than broader transparency context. |
| 45 | Jang et al. (2024) Knowledge of automated journalism moderates evaluations of algorithmically generated news | Experiment | Strengths: randomized experiments; multiple story topics; tests both attribution and knowledge/anthropomorphism framing. Limitations: sports-only context; likely label-based manipulation (unclear if text itself was AI-written); generalizability to other news beats and real-world disclosures may be limited. |
| 46 | Krausová & Moravec (2022) Disappearing Authorship: Ethical Protection of AI-Generated News from the Perspective of Copyright and Other Laws | Qualitative | Strengths: combines legal/ethical analysis with primary qualitative data from practitioners; includes comparison across newsroom types. Limitations: small, single-country professional sample; not an audience study; findings may not generalize beyond Czech contexts or beyond the studied organizations. |
| 49 | Kreps et al. (2022) All the News That’s Fit to Fabricate: AI-Generated Text as a Tool of Media Misinformation | Unclear/other | Strengths: multi-experiment design; systematic variation in model size and disclosure; realistic news-like stimuli. Limitations: MTurk convenience samples; GPT-2 (pre-ChatGPT era) may differ from current LLM outputs; some disclosure details rely on appendix; publication status may differ from SSRN version (later journal publication possible). |
| 51 | Schulz et al. (2022) User Experience Design for Automatic Credibility Assessment of News | Survey | Strengths: experimentally tests interface-level explanations; uses multiple news items and measures both performance and perceptions. Limitations: domain-specific (COVID-19); potential over-reliance/automation bias; sample/recruitment details and ecological validity depend on full deployment context. |
| 53 | Zoizner et al. (2025) Can AI-Attributed News Challenge Partisan News Selection? Evidence from a Conjoint Experiment | Experiment | Strengths: preregistered; large, well-powered sample; ecological design mimicking social media; multiple cues manipulated simultaneously. Limitations: forced-choice design (no ‘avoid news’ option); AI role described generally (not task-specific); fictitious AI outlet may affect realism; mechanisms harder to isolate in conjoint designs. |
| 54 | Spinde et al. (2025) Enhancing media literacy: The effectiveness of (Human) annotations and bias visualizations on bias detection | Unclear/other | Strengths: large samples, preregistration, explicit generalization test after visualization removal. Limitations: Prolific sampling and selected topics; AI labels are classifier-based (imperfect agreement with human labels). |
| 55 | Danry et al. (2025) Deceptive Explanations by Large Language Models Lead People to Change their Beliefs About Misinformation More Often than Honest Explanations | Experiment | Strengths: preregistration and large dataset. Limitations: simulated interaction setting; model is GPT-3-era; results may depend on curated deceptive prompting and selected statements. |
| 56 | Govers et al. (2025) Feeds of Distrust: Investigating How AI-Powered News Chatbots Shape User Trust and Perceptions | Unclear/other | Strengths: realistic 'news+chat' use case and multi-topic within-subject design. Limitations: modest N, US-only, de-identified sources reduce ecological source cues; chatbot behavior depends on prompt design. |
| 57 | La-Rosa & SANDOVAL-MARTIN (2024) Artificial intelligence versus journalists: The quality of automated news and bias by authorship using a Turing test | Experiment | Single-topic, two-text stimuli; young and journalism-heavy sample; early pandemic timing. Strength: controlled presentation removes confounds from outlet design cues. |
| 58 | Chen et al. (2025) Generative AI in the News: The Impact of Framing on Public Attitude and Engagement | Unclear/other | Observational/archival (no causal inference); limited to selected outlets and Facebook platform; timeframe ends March 2024. |
| 59 | García-Marín et al. (2025) “Everything is believable”. Credibility of disinformation produced by using AI and the perception of Spanish communication students | Survey | Strength: classroom control reduces cheating. Limitations: student sample and six short items; outcomes may depend on chosen prompts and topics; no disclosure condition only. |
| 60 | Gherheş et al. (2025) AI vs. Human-Authored Headlines: Evaluating the Effectiveness, Trust, and Linguistic Features of ChatGPT-Generated Clickbait and Informative Headlines in Digital News | Unclear/other | Student-only sample and Romanian context; headline-only evaluation (no article reading); no-disclosure design isolates style effects but cannot estimate labeling effects. |
| 61 | Velásquez-Salamanca et al. (2025) Interpretation of AI-Generated vs. Human-Made Images | Unclear/other | Stimulus set intentionally imbalanced (3:1 AI:human). Recruitment/country details limited; outcomes may depend on tool versions/prompts and chosen human-image repository. \| OA link: https://www.mdpi.com/2313-433X/11/7/227 (MDPI Journal of Imaging (open access)) |
| 62 | Wu & Li (2024) Journalists' Technological Trust and Willingness to Use Generative AI: A Perspective Based on Risk Perception Theory | Survey | Focuses on professionals’ adoption (not audience perceptions of AI-authored news). Sampling frame and country context are not fully specified in the paper beyond author affiliation. |
| 64 | Cools & Diakopoulos (2024) Uses of Generative AI in the Newsroom: Mapping Journalists’ Perceptions of Perils and Possibilities | Unclear/other | Strength: rich qualitative mapping of concrete newsroom GenAI use cases; cross-national (NL/DK) perspectives. Limitations: small convenience sample focused on self-identified early adopters; self-report; findings may not generalize to non-adopters or other media systems. |
| 65 | Jia & Johnson (2021) Source Credibility Matters: Does Automated Journalism Inspire Selective Exposure? | Experiment | Strength: factorial design manipulating AI vs human byline with attitude-congruence and topic; includes behavioral selection (selective exposure) and mediation. Limitations: MTurk convenience sample; uses one algorithm-provider framing (“Automated Insights”) and a fictional news site; limited to two issues (gun/abortion). |
| 69 | Dobber et al. (2025) A Beacon of Trustworthiness in a Sea of Disinformation: Does News Coverage About the Dangers of Generative AI Cause People to Flock to Journalism? | Experiment | Strengths: preregistered design; multiple frame manipulations; panel recruitment with demographic balance. Limitations: single-country study; single brief exposure; framed stimuli differed in length; relies on self-reported intentions and trust perceptions. |
| 70 | Yeste-Piquer et al. (2025) What If I Prefer Robot Journalists? Trust and Objectivity in the AI News Ecosystem | Unclear/other | Strengths: rich qualitative evidence; deliberately varied age and AI familiarity; considers both text and video manipulation contexts. Limitations: small, city-based sample (Spain); focus-group dynamics/social desirability; findings not generalizable and no causal test of labels/bylines. |
| 73 | DeVerna et al. (2024) Fact-checking information from large language models can decrease headline discernment | Experiment | Large preregistered experiment; key limitation for synthesis: intervention is AI fact-checking, not AI-generated journalism authorship per se (classify as AI-augmented credibility intervention). |
| 74 | Sun et al. (2024) Public Perceptions and Attitudes Towards the Application of Artificial Intelligence in Journalism: From a China-based Survey | Survey | Self-selected online panel sample; measures reflect general attitudes rather than direct credibility/trust judgments of a specific AI-generated news story. |
| 75 | Cloudy et al. (2023) The Str(AI)ght Scoop: Artificial Intelligence Cues Reduce Perceptions of Hostile Media Bias | Experiment | Single-issue, single-message stimulus; Prolific partisan sample may limit generalizability beyond U.S.-political social media contexts. |
| 77 | Jeng et al. (2024) Emotional Reframing of Economic News using a Large Language Model | Unclear/other | Potential confound: framing condition is confounded with source of reframing (GPT-4 vs journalist) unless disclosure/source was experimentally controlled; recruitment/country details need confirmation from full method section. |
| 80 | Gong (2023) AI voices reduce cognitive activity? A psychophysiological study of the media effect of AI and human newscasts in Chinese journalism | Unclear/other | Small lab sample; recruitment source unspecified; psychophysiological measures strengthen inference about cognitive processing but limit generalizability. |
| 81 | Kim & Kim (2020) A Decision-Making Model for Adopting Al-Generated News Articles: Preliminary Results | Survey | Small purposive professional sample; contributes contextual/organizational perspective rather than audience credibility effect estimates. |
| 84 | Lermann Henestrosa & Kimmerle (2024) The Effects of Assumed AI vs. Human Authorship on the Perception of a GPT-Generated Text | Experiment | Preregistered (aspredicted). Strong internal validity (identical stimulus text; large N). External validity may be limited to German-speaking Prolific sample and single science topic. |
| 85 | Liu & Wei (2019) Machine Authorship In Situ: Effect of news organization and news genre on news credibility | Experiment | MTurk convenience sample; multiple exclusion steps (manipulation checks); stimulus texts adapted from existing reports; generalizability limited to US online workers and selected topics. |
| 86 | Fernández-Barrero & Serrano-Martín (2025) Are the Media Transparent in Their Use of AI? Self-Regulation and Ethical Challenges in Newsrooms in Spain | Survey | Small survey sample (N=50) and interviews (n=8); self-report and potential social desirability; Spain-only context; descriptive (no causal tests). |

Note. In Table E1, ID corresponds to the included-study record_id used in Appendix A (sheet “included for analysis fulltext”) and Appendix B (sheet “included_fulltext_unique”). The PRISMA master list uses prisma_record_id.
